# Interfacial Control in Cu–MXene Hybrids Enables Selective NOx‐to‐NH_3_ Electroconversion: A Critical Review

**DOI:** 10.1002/advs.76280

**Published:** 2026-06-28

**Authors:** Hafiz Muhammad Adeel Sharif, Gechuanqi Pan, Yuwei Wang, Yasir Abbas, Asif Mahmood, Changping Li

**Affiliations:** ^1^ Research Center for Eco‐Environmental Engineering Dongguan University of Technology Dongguan China; ^2^ School of Chemical Engineering and Energy Technology Dongguan University of Technology Dongguan China; ^3^ Interdisciplinary Research Center for Membranes and Water Security King Fahd University of Petroleum & Minerals Dhahran Saudi Arabia; ^4^ School of Mathematical and Physical Sciences Faculty of Science University of Technology Sydney Sydney New South Wales Australia; ^5^ Institute of Sustainable Industries and Liveable Cities Victoria University Melbourne Victoria Australia

**Keywords:** Cu‐catalysts, electrocatalysis, MXenes, NH_3_ synthesis, NO_x_ reduction

## Abstract

Electrochemical conversion of nitrogen oxides (NO_x_, mainly NO and NO_2_) to ammonia (NH_3_) transforms a pollutant into a valuable chemical, providing a direct link between emissions control and low‐carbon NH_3_ production under mild conditions. However, NO_x_ reduction competes with hydrogen evolution (HER) and can diverge to N_2_/N_2_O, while many catalysts restructure under bias, making results difficult to compare and design rules hard to extract. Cu‐MXene hybrids are emerging as a compelling platform because the 2D interface can be engineered to control adsorption, electron (e^−^) transfer, and local proton (H^+^) activity through Cu speciation (single sites, clusters, oxides, reconstructed surfaces) and MXene terminations/defects. This critical review summarizes the interfacial control of these catalysts to achieve selective NO_x_‐to‐NH_3_ electroconversion. The review provides a comprehensive analysis of intermediates and performance, quantifying using device‐relevant metrics (Faradaic efficiency, NH_3_ yield/partial current, energy efficiency, durability, and selectivity against N_2_/N_2_O and HER). Special emphasis is placed on reactor design, including flow‐cell and gas‐diffusion electrode (GDE) configurations, which enhance mass transport, stabilize the three‐phase boundary, and enable long‐term operational stability. Finally, the review emphasizes the use of in situ/operando methods to investigate the reaction mechanisms, outlines design rules and scale‐up priorities for practical NO_x_ upcycling into green ammonia.

## Graphical abstract

Cu‐MXene interfacial catalysts provide a promising platform for electrochemical NOx upcycling to ammonia. The synergy between Cu active sites and conductive, surface‐tunable MXene supports promotes NOx adsorption, intermediate stabilization, and selective NH_3_ formation while suppressing competing hydrogen evolution, offering a route toward sustainable nitrogen recycling and pollution‐to‐value conversion.

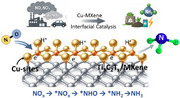

## 1 Introduction

Ammonia (NH_3_) is a foundation chemical for fertilizers and a key feedstock for many industries, and it is increasingly discussed as a practical carbon‐free energy carrier because it is easier to store and transport than hydrogen. Up till now, almost all NH_3_ is still produced by the Haber–Bosch process, which couples N_2_ and H_2_ under high‐temperature and pressure and depends heavily on fossil‐derived hydrogen [1, 2]. This makes NH_3_ production both energy intensive and carbon intensive, with widely reported estimates placing it at around a few percent of global energy use and roughly ∼1%–2% of annual CO_2_ emissions [3, 4]. Decarbonising this supply chain is therefore a central challenge for both food security and energy transition. Over the last decade, major efforts have been directed toward green NH_3_ synthesis routes that operate under mild conditions and use renewable electricity with abundant inputs such as air and water [5]. Electrochemical approaches are particularly attractive because they offer modular operation and direct coupling to intermittent renewable power. However, the most studied route, electrochemical N_2_ reduction (NRR), still faces fundamental bottlenecks including poor N_2_ solubility, slow kinetics, and strong competition from hydrogen evolution, which together limit NH_3_ selectivity and formation rates in many systems [6].

**FIGURE 1:**
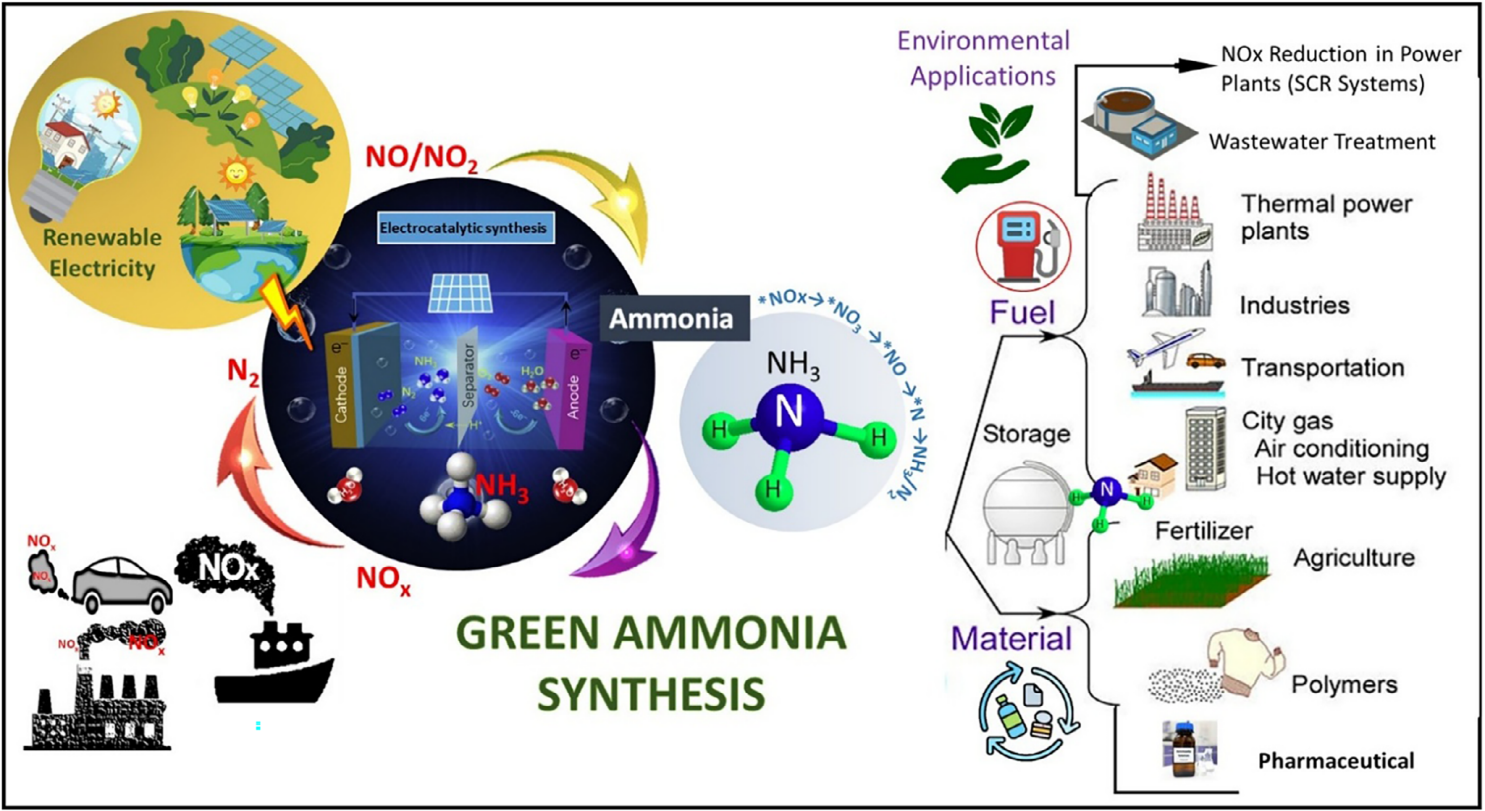
NH_3_ synthesis by the electrochemical route provides numerous advantages for a carbon‐free energy future.

A complementary pathway gaining momentum is electrochemical NO_x_‐to‐NH_3_ conversion, where NO and NO_2_ serve as reactive nitrogen feedstocks (Figure 1). This concept is compelling for two reasons. First, NO_x_ species are generally easier to activate than N_2_, shifting the chemistry from breaking an exceptionally stable N≡N bond to controlling multi‐step hydrogenation of N‐O intermediates under electrochemical bias. Second, it offers a rare “dual benefit” framework: NO_x_ is a harmful pollutant from combustion, power generation, and industrial activity, linked to smog formation, acidification, and adverse respiratory outcomes, while NH_3_ is a valuable product [7, 8, 9, 10]. Converting NO_x_ into NH_3,_ therefore, couples pollution mitigation with resource recovery and can be driven by renewable electricity under ambient conditions, as shown in Figure 1. However, the electrochemical NO_x_ reduction involves multiple competing pathways and selectivity issues, which can lead to undesired products (N_2_ or N_2_O), while hydrogen evolution can consume a large fraction of electrons [11]. Chorkendorff and colleagues methodically investigated the lithium/calcium‐mediated NO reduction reaction (NORR) to address the shortcomings of conventional electrochemical NORR, which significantly increased the rate of FE and NH_3_ synthesis for NORR to generate NH_3_ [1, 2, 12]. In addition, Zhang's group developed a jet plasma oxidation of N_2_ to NO_2_ followed by an electrochemical reduction step to convert the N_2_ into NH_3,_ where they studied lithium reactivity and the solid‐electrolyte interphase via cryo‐electron microscopy, to achieve lithium‐mediated NO_x_RR [13, 14]. However, because of competing reactions and the large dissociation energy of N≡N, the NH_3_ synthesis FE, yield rates, and cost of electrochemical NO_x_ (eNO_x_RR) are still far from industrial requirements. Therefore, it hinges on catalyst design that can stabilize the right intermediates, suppress side reactions, and maintain high current density and stability in realistic device formats, including gas‐fed electrodes and flow systems [15, 16, 17].

**FIGURE 2:**
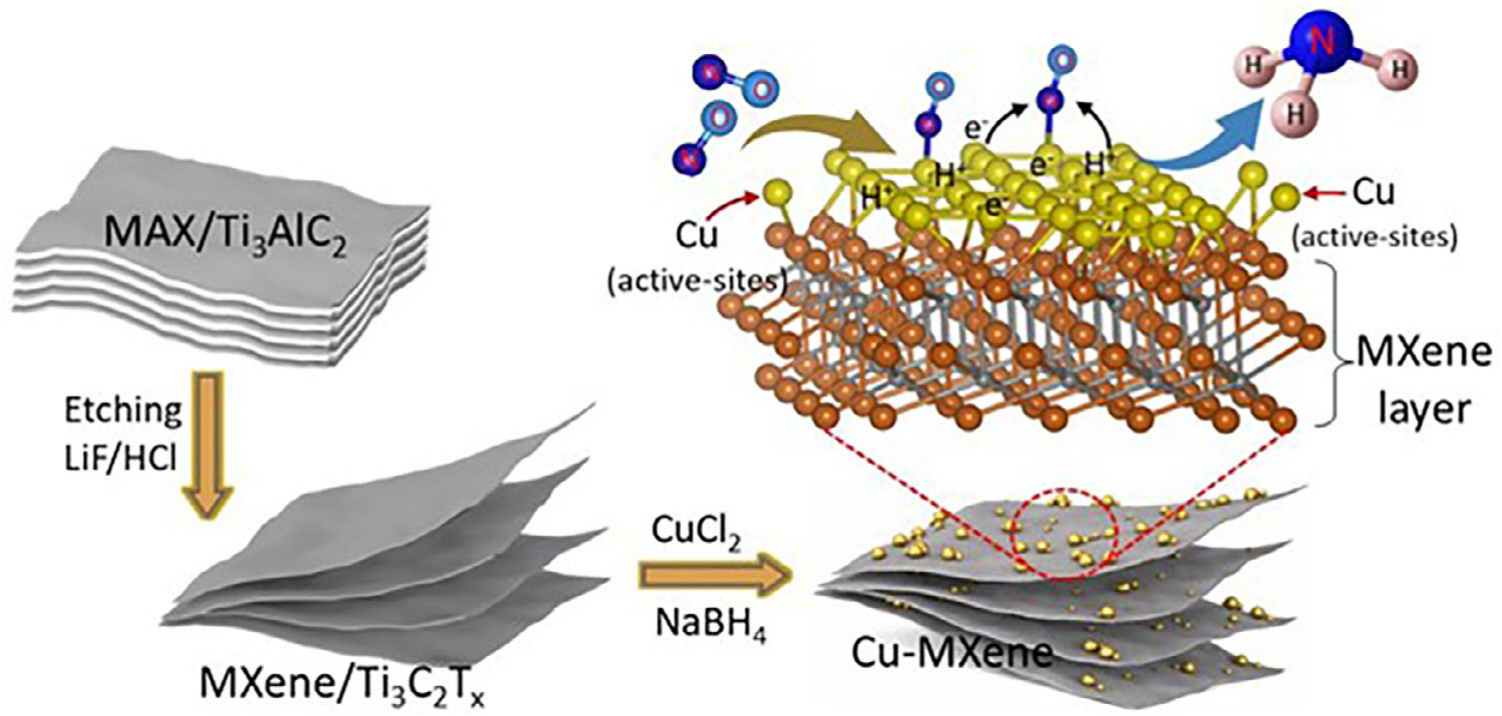
Schematic illustration of Cu–MXene synthesis and its catalytic role. Ti_3_AlC_2_ is etched to form Ti_3_AlC_2_/MXene, followed by Cu deposition, where the interface enhances NO_x_ adsorption and promotes efficient NH_3_ formation.

Among the catalyst classes explored for NO_x_ electroreduction, copper (Cu) stands out because it is earth‐abundant, electrically conductive, and capable of binding nitrogen‐oxygen intermediates, thereby favoring stepwise hydrogenation toward NH_3_ under suitable conditions [18, 19, 20]. Still, Cu‐only catalysts often face trade‐offs between activity, selectivity, and durability, driven by surface reconstruction, limited control over intermediate adsorption energies, and sensitivity to electrolyte and mass‐transport effects. This has pushed the community toward hybrid architectures where Cu is coupled to a second material that can tune interfacial charge transfer and local reaction energetics [21, 22]. MXenes, a family of conductive 2D transition‐metal carbides/nitrides with tunable surface terminations, have emerged as particularly promising platforms because they offer high accessible surface area, strong anchoring sites for metals, and adjustable electronic structure and wettability [23, 24]. In Cu‐MXene composites, these features can increase the density of catalytically active Cu sites, stabilize active‐state Cu species, and modulate adsorption of key NO_x_‐derived intermediates, collectively improving NH_3_ selectivity and operational stability. Recent years have seen tremendous interest in these types of catalysts, reporting innovative strategies to boost the electrochemical NH_3_ synthesis [19, 25, 26, 27]. Therefore, this review focuses on Cu‐MXene‐based 2D catalysts for high‐performance NO_x_‐to‐NH_3_ electroconversion, summarising mechanistic understanding, structure‐property relationships, device‐relevant performance metrics, and design principles needed to move from proof‐of‐concept studies toward practical NO_x_ upcycling and green ammonia production [28]. To provide a fundamental understanding of Cu‐MXene catalyst construction and functionality, the preparation route and catalytic mechanism are illustrated in Figure 2.

**FIGURE 3:**
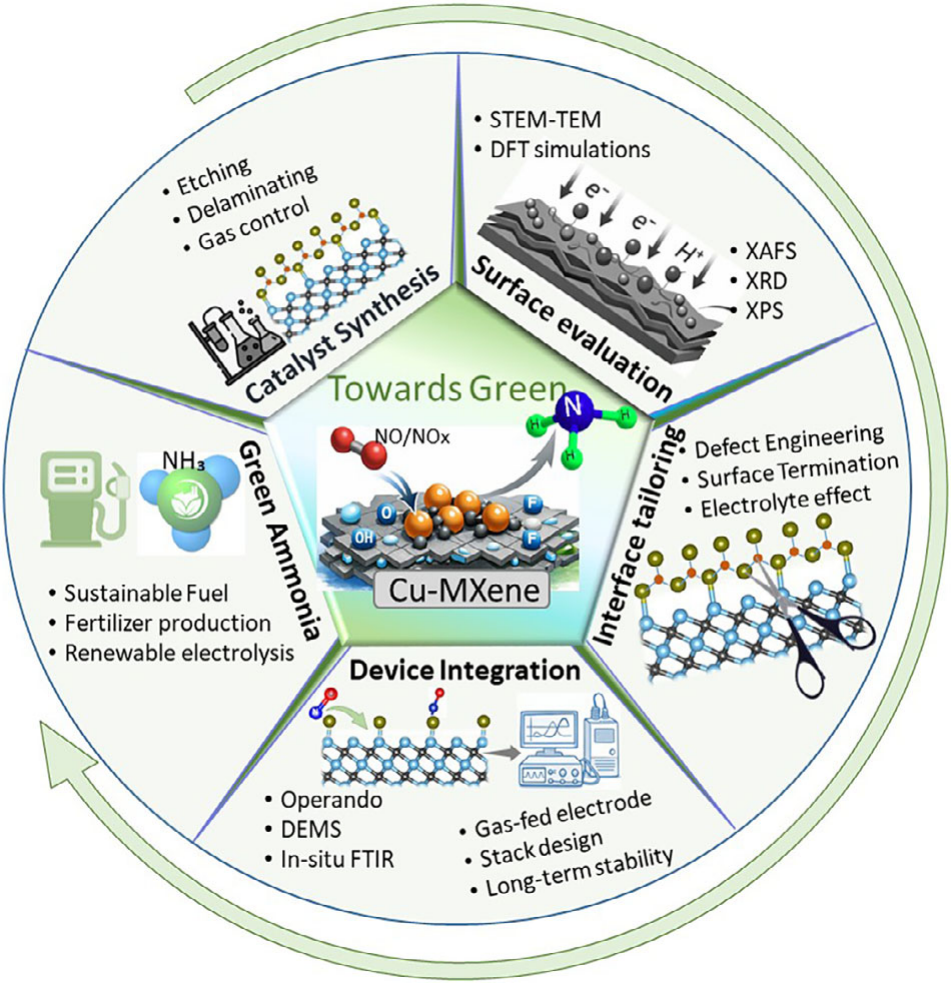
Timeline development from basic to advancement in NH_3_ synthesis and minimising carbon contents, a sustainable green strategy.

Over recent years, the development of eNO_x_RR catalysts has advanced from conventional metal‐based systems to sophisticated heterostructured materials like Cu‐MXene. This evolution has steadily improved catalytic activity, NH_3_ selectivity, and operational stability by optimizing interfacial properties and reaction pathways. Key milestones in this progression are summarized in the timeline shown in Figure 3.

## 2 Major Challenges in eNOxRR

**FIGURE 4:**
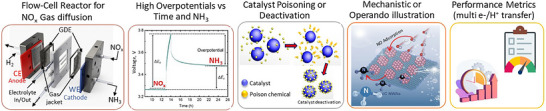
Schematic illustration of challenges in Cu‐MXene‐based electrocatalytic systems conversion of NO_x_ into NH_3_, highlighting flow cell reactor configuration better mass transfer and efficiency, overpotential, catalyst deactivation, interfacial hydrogenation pathways, and key performance limitations.

Electrochemical reduction of nitrogen oxides to ammonia (eNOxRR) is attractive because it can, in principle, couple NO_x_ remediation with green NH_3_ production under mild conditions. However, the reported systems often suffer from intertwined limitations in (i) reactant delivery and adsorption, (ii) energy efficiency and overpotential, (iii) catalyst stability and poisoning, and (iv) inconsistent benchmarking and quantification. These barriers directly control NH_3_ selectivity, achievable current density, and long‐term durability, which are key requirements for practical deployment. Figure 4 summarizes the major challenges for Cu‐MXene‐based eNO_x_RR systems, and the following sections discuss each bottleneck in a mechanistic, design‐oriented manner. This section provides a detailed summary of the challenges in eNO_x_RR.

### 2.1 NO_x_ Adsorption and Mass Transfer

eNO_x_RR is initiated by the access of NO_x_ to the electrode and the formation of surface‐bound intermediates. Inadequate adsorption or poor reactant supply lowers surface coverage of productive intermediates and shifts electrons toward competing reactions, particularly HER. At the mechanistic level, NO can adsorb through either N or O atoms, where its adsorption can strongly influence the sequence of deoxygenation and hydrogenation events [29, 30, 31]. The catalyst must therefore satisfy a Sabatier‐type balance across a set of intermediates rather than a single adsorbate: overly strong binding increases surface residence time and site blocking (apparent poisoning), whereas weak binding promotes premature desorption and incomplete hydrogenation, often increasing soluble NO_x_‐derived species and decreasing NH_3_ selectivity [32, 33].

Cu is widely explored because it interacts favorably with NO_x_‐derived intermediates and can enable stepwise hydrogenation under suitable potentials [34, 35]. However, bare Cu surfaces can also be susceptible to excessive coverage by strongly bound NO_x_‐related species, thereby reducing the density of free active sites during extended operation and contributing to catalyst stability losses [36]. As a result, catalyst design often focuses on tuning adsorption energies and preferred binding modes through electronic‐structure modulation (e.g., alloying or dopants), defect engineering, and interface construction [37, 38, 39]. In Cu‐MXene catalysts, the support is not merely a passive scaffold: Cu‐MXene coupling and MXene surface terminations can reshape local e^−^ density, stabilize particular Cu states, and adjust the adsorption landscape of key intermediates, providing additional control over pathway selectivity [20, 27].

In many practical configurations, mass transfer becomes equally limiting, especially for gas‐fed NO/NO_2_ due to low solubility in aqueous electrolytes. Under these conditions, apparent activity is often governed by reactant flux to the three‐phase boundary rather than intrinsic kinetics. Gas diffusion electrodes (GDEs), membrane electrode assembly reactors (MEA), and flow cells reactors are increasingly adopted to raise NO_x_ utilisation and maintain higher current densities [40]. Electrode architecture (porosity, hydrophobic/hydrophilic balance, ionomer distribution, and catalyst‐layer thickness) then directly controls local NO_x_ concentration, intermediate residence time, NH_3_ and oxygenated product removal. MXene‐based electrodes add specific transport risks, including restacking and restricted interlayer diffusion, as well as flooding that blocks gas pathways, all of which lower accessible surface area and degrade performance [41, 42]. Flow‐cell reactors provide continuous reactant and electrolyte supply, stabilizing the three‐phase boundary and mitigating NO_x_ solubility limitations, while enabling higher current densities and reproducible NH_3_ selectivity. The details about flow cells reactors also given in Table 1. Achieving high‐rate eNO_x_RR requires co‐optimisation of surface chemistry (adsorption balance) and transport design (stable three‐phase delivery), rather than treating them independently.

**TABLE 1:** Overview of catalysts and flow‐cell/GDE reactors for electrochemical NO_x_ reduction, showing feed current density, selectivity, and key operational highlights.

Catalyst	Reactor Type	Feed	Current Density	Product / Selectivity	Key Notes	References
Cu, Pd	Gas‐fed 3‐compartment flow cell	25%–100% NO (gas)	Up to 400 (mA/cm^2^)	NH_3_/∼100% FE	High‐rate ambient eNO_x_RR; pH and NO coverage critical	[43]
Fe/C (60 wt.% Fe)	Flow cell with GDE	1%–10% NO (gas)	Upto 165 (mA/cm^2^)	NH_3_/50%–96% FE	High NH_3_ yield; stable 70 h; GDE improves mass transport	[44]
Cu foam	Gas Diffusion Electrode (GDE)	0.1% NO (gas)	50 (mA/cm^2^)	NH_3_/95% FE	Efficient NO mass transport; continuous catholyte flow	[45]
Cu(OH)_2_ nanoelectrode	Zero‐gap flow reactor	100 mg‐N·L^−1^ NO_3_^−^ (solution)	15 (mA/cm^2^)	N_2_/80% FE	Compact‐electrode; full NO_3_^−^ conversion; wastewater relevant	[46]
Co foam cathode,	3D Flow cell with serpentine channel	0.1 m NO_3_^−^ (solution)	50 (mA/cm^2^)	NH_3_ ∼1.4 mol·m^−3^·s^−1^	Serpentine channel; high NH_3_ production; modeling validated	[47]
Cu foam cathode,	Continuous dual‐channel cell	0.1 m NO_3_^−^ (solution)	50 (mA/cm^2^)	NH_3_ main product; NO_2_ intermediate	Continuous flow; NO_3_^−^→NO_2_^−^→NH_3_; rate & area affect yield	[48]
Cu‐Ti	Flow‐through hollow fiber electrode	10% NO (gas)	50 (mA/cm^2^)	NH_3_/∼90% FE	High NH_3_ production; stable and practical flow‐cell scalability	[45]
Cu Pd_1_ single‐atom alloy	Flow cell	NO (feed gas)	210 (mA/cm^2^)	NH_3_/∼85.5% FE	Flow cell; Pd_1_Cu single‐atom, high stability	[22]

### 2.2 High Overpotential

Even when NO_x_‐to‐NH_3_ conversion is thermodynamically feasible, practical eNO_x_RR often demands a substantial overpotential, directly compromising energy efficiency. The extra voltage arises from a combination of (i) kinetic barriers associated with N─O bond breaking and stepwise hydrogenation, (ii) ohmic losses through the electrolyte, membrane, and catalyst layer, and (iii) concentration polarisation when NO_x_ delivery (or product removal) is transport limited. Pushing to more negative potentials accelerates HER and consumes protons/electrons that would otherwise contribute to NH_3_, lowering FE_(NH3)_ and increasing energy loss per mole of product [15, 49].

Mechanistically, the overpotential problem reflects the reaction network complexity. Selective NH_3_ formation requires coordinated control of multiple coupled H^+^/e^−^ transfers and the timely removal of oxygen from NO_x_‐derived intermediates. The catalyst must stabilize productive intermediates while avoiding surfaces that either bind them too strongly (site blocking) or too weakly (premature desorption). At the same time, it must suppress HER on the same interface [50, 51]. These constraints explain why many systems show a trade‐off between NH_3_ selectivity and NH_3_ production rate: conditions that enhance j often reduce FE, while conditions that maximize FE may operate at low *j*. Recent catalyst engineering has shifted NH_3_ formation to less negative potentials by increasing the accessible active‐site density and tuning intermediate binding landscapes [49]. Still, device‐level gains also require minimising resistive losses and reducing transport‐driven polarisation. Ultimately, reducing overall cell voltage will depend on co‐optimising catalyst chemistry and electrode/cell architecture [16].

### 2.3 Catalyst Deactivation or Poisoning

**FIGURE 5:**
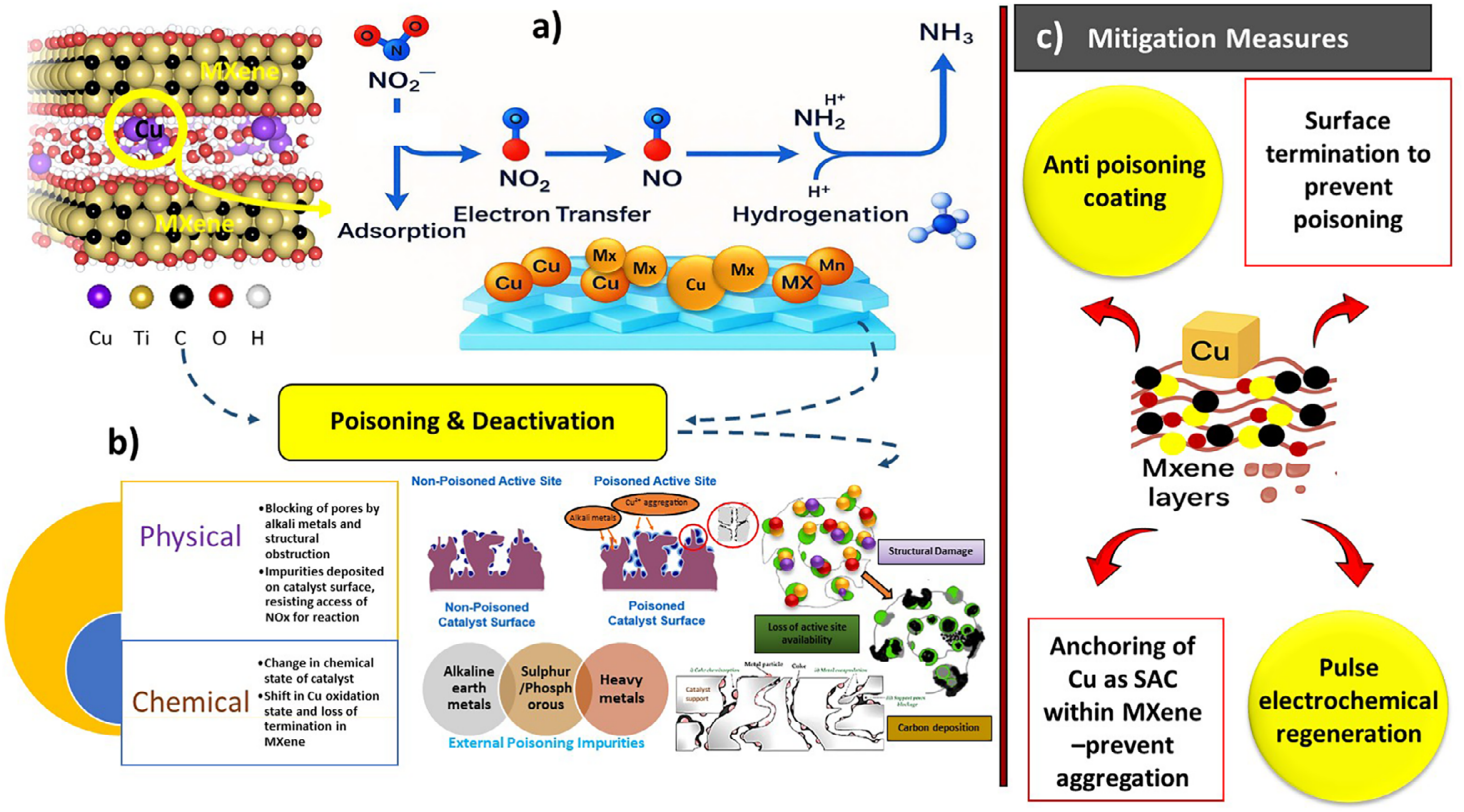
(a) Layout of Cu‐Mxene and its adsorption mechanism, (b) poisoning and deactivation of Cu‐Mxene surface, and (c) mitigation measures applied for the promising design principles for fabrication robust Cu‐MXene electrocatalysts, reprinted with permission from ACS Nano [60].

A major limitation in eNO_x_RR is the progressive loss of catalytic performance during operation. Activity and NH_3_ selectivity often drop with time because the identity and availability of active sites change under bias. One intrinsic route is intermediate‐induced site blocking. eNOxRR proceeds through strongly adsorbed N─O and N─H intermediates, and species such as *NO and *NH_x_ can accumulate on the surface long enough to suppress turnover. This effectively reduces the number of free sites for NO_x_ activation and increases sensitivity to HER, particularly on Cu surfaces that can stabilize nitrosyl‐like adsorbates under reducing conditions [17, 52]. A second route is impurity‐driven deactivation. Spectator ions and contaminants introduced by electrolytes, membranes, or feeds can block pores, alter wettability, and alter the chemical state (Figure 5a). In Cu‐containing systems, alkali and alkaline‐earth species can shift Cu coordination and speciation, promoting transformation of catalytically relevant Cu sites into less active CuO_x_‐like forms and reducing accessible surface area through pore blockage [53, 54].

For MXene‐based electrodes, additional failure modes include scaffold oxidation, rearrangement or loss of surface terminations (─O/─OH/─F), and restacking/interlayer collapse, which reduce conductivity and accessible interfacial area [55]. These effects are especially damaging because Cu‐MXene performance depends on maintaining a well‐defined interface [28, 56]. For Cu‐MXene catalysts, deactivation can be viewed in two buckets: physical (restacking, flooding, pore blockage, transport disruption) and chemical (strong intermediate adsorption, Cu‐state drift/reconstruction, MXene oxidation and termination changes; Figure 5b. These challenges must be addressed to achieve efficient eNO_x_RR performance using MXenes‐based catalytic systems [57, 58].

Progress in eNO_x_RR is often difficult to assess because performance is not always reported. This is non‐trivial for NO_x_‐to‐NH_3_ because the reaction is multi‐electron/multi‐proton, and the electron requirement depends on the feed: NO‐to‐NH_3_ is a 5e^−^ process, nitrite‐to‐NH_3_ is 6e^−^, and nitrate‐to‐NH_3_ is 8e^−^ (acidic equivalents). As a result, FE and apparent rates can shift simply due to feed identity (gas vs. dissolved), speciation, and local interfacial pH, even when the catalyst is unchanged. Therefore, the studies should include FE_(NH3)_, partial current density to NH_3_ (*j*_NH3_), NH_3_ formation rate (area‐normalized and, when appropriate, mass‐normalized), and an energy metric such as cell voltage or energy efficiency under clearly defined conditions [39, 53].

Analytical reliability is equally critical. NH_3_ quantification is vulnerable to background contamination, nitrogen‐containing impurities in electrolytes and labware, and adsorption/desorption or crossover associated with membranes and cell components. Strong studies therefore include rigorous blanks and controls (open‐circuit, inert feed, no catalyst) and, where feasible, isotopic verification to confirm the nitrogen source. Finally, durability must be evaluated under device‐relevant conditions: retention of FE_(NH3)_ and *j*NH_3_ over time at fixed current/potential, alongside post‐test characterisation to verify that Cu speciation, MXene terminations, and electrode architecture remain stable (Figure 5c). Without consistent benchmarking and robust quantification, apparent gains may reflect artefacts or transport effects rather than true catalytic improvement [58, 59, 60].

### 2.4 Challenges in Performance Evaluation and Benchmarking

While the preceding sections outline the major challenges in eNO_x_RR, the following discussion focuses on the key performance evaluation metrics. These parameters, such as FE, current density, NH_3_ yield, and durability, are critical for benchmarking catalysts but do not constitute intrinsic limitations in themselves. Evaluating electrocatalysts for NO_x_ reduction to NH_3_ is not straightforward, largely because the reaction unfolds through complex multi‐electron and proton‐coupled steps at interfaces that are continuously evolving under operating conditions. Unlike simpler redox reactions, eNO_x_RR follows a highly interconnected pathway in which sequential H^+^/e^−^ transfers, shifting interfacial speciation, and competing adsorption processes occur simultaneously. In practice, this means that widely used performance metrics such as FE, partial current density (*j*), overpotential (η), yield rate, selectivity, and durability cannot be treated as purely intrinsic properties of the catalyst. Instead, they often reflect the local reaction environment just as much as the catalyst itself. As a result, comparing different systems can be less straightforward than it appears, and reported performance differences should be interpreted with some caution [61, 62].

A key challenge stems from the intrinsic complexity of reaction pathways in NO_x_ reduction. The conversion to NH_3_ does not proceed along a single well‐defined route; rather, it involves a network of parallel and sequential steps, mediated by intermediates such as *NO, *NOH, *NHOH, and *NH_x_. These species compete for both active sites and available hydrogen at the interface, making the overall process highly sensitive to local conditions. In practice, even slight variations in H^+^ activity, interfacial electric fields, or adsorption energetics can alter the distribution of reaction flux, often redirecting electrons toward side products such as N_2_ or N_2_O. This sensitivity makes FE_(NH3)_ difficult to interpret straightforwardly. It does not solely reflect intrinsic selectivity, but rather captures the combined influence of competing pathways and transport effects operating at the interface. Interpreting activity metrics is not always straightforward, as the measured current density can be influenced by both intrinsic catalytic activity and mass transport effects. In systems like gas‐diffusion electrodes, improved reactant supply raises *j*_(NH3)_ without a big change in active‐site performance, bringing normalisation essential for significant comparison [62].

Overpotential introduces an additional level of complexity, as it captures several energetic losses, including barriers associated with N─O bond cleavage, interfacial charge‐transfer resistance, and concentration polarisation. In practice, the potentials required to activate NO_x_ reduction tend to overlap with those driving HER, making balancing activity and selectivity challenging. Pushing to more negative potentials can increase reaction rates but also favor hydrogen production, lowering FE. Managing this balance relies on controlling interfacial hydrogen coverage and adsorption competition, which are strongly influenced by the catalyst's electronic structure and the local electrolyte environment. Selectivity remains difficult to assess, largely because HER is dominant in aqueous media. Most catalyst surfaces favor H^+^ reduction, leading to preferential H* coverage that limits NO_x_ adsorption. This competition shifts with potential, pH, and electrolyte conditions, which often complicates reproducibility across studies [63, 64].

Reliable product quantification remains a persistent challenge. Measuring NH_3_ at low concentrations is particularly prone to interference from environmental contamination, nitrogen‐containing impurities, and membrane crossover. Adsorption‐desorption behavior and interactions with the electrolyte can further skew concentration measurements, sometimes giving an inflated impression of performance. In practice, careful control experiments and isotopic labeling are necessary; without such precautions, it is difficult to distinguish genuine catalytic activity from experimental artifacts [65]. Finally, evaluating durability is not straightforward, as catalyst surfaces tend to evolve continuously under electrochemical bias. Structural reconstruction, changes in oxidation state, intermediate‐induced site blocking, and even support degradation, particularly in MXene‐based systems, can gradually reshape the active‐site landscape during operation. These effects often go unnoticed in short‐term tests, which can make initial performance appear more stable than it actually is. Capturing such behavior requires operando approaches that link microkinetic insights with interfacial characterisation and time‐resolved measurements under realistic conditions.

### 2.5 Ideal Catalyst Design

An ideal eNO_x_RR catalyst is defined by a simultaneous balance of selectivity, activity, energy efficiency, and long‐term stability under applied conditions. Achieving high FE_(NH3)_ alone is insufficient if it occurs at low current density or requires strongly negative potentials. In practice, the catalyst should deliver high *j*_NH3_ at low overpotential and maintain performance over long operation. This balance is difficult due to the underlying reaction involving multi‐step H^+^/e transfer, competing pathways, and strong coupling with interfacial hydrogen dynamics.

The catalyst design requirement must provide balanced binding across NO_x_ reduction and key intermediates (*NO_x_, *NO, *NOH, *NH_x_). It should be strong enough to stabilize for activation and hydrogenation, but weak enough to avoid site blocking. At the same time, it must suppress HER while still enabling fast H^+^/e^−^ transfer to NO_x_‐derived intermediates. The catalyst should also provide a high density of accessible active sites within an electrode architecture that avoids transport bottlenecks. Furthermore, it must maintain a stable active state under bias, resisting reconstruction, oxidation‐state drift, leaching, and support degradation. In addition, tolerance to impurities and spectator ions, which can otherwize alter surface chemistry and accelerate deactivation [33, 66].

From a practical point of view, Cu‐MXenes are particularly attractive because they offer an interface where these targets can be engineered rather than traded off. Cu provides reactive centers for NO_x_ activation and hydrogenation, while MXenes provide a conductive 2D scaffold with tunable terminations and anchoring sites that can stabilize Cu dispersion and reshape interfacial energetics. The central design strategy thus lies in precise interface control, including tailoring Cu speciation and anchoring, optimising metal‐support interactions, and engineering termination/defect chemistry. In parallel, aligning electrode microstructure with transport demands is compulsory to sustain selective NO_x_‐to‐NH_3_ conversion at device‐relevant conditions [67, 68].

## 3 Efficiency Governing Aspects for eNO_x_RR

### 3.1 Role of Cu Entities and Morphology of MXene Surface

**FIGURE 6:**
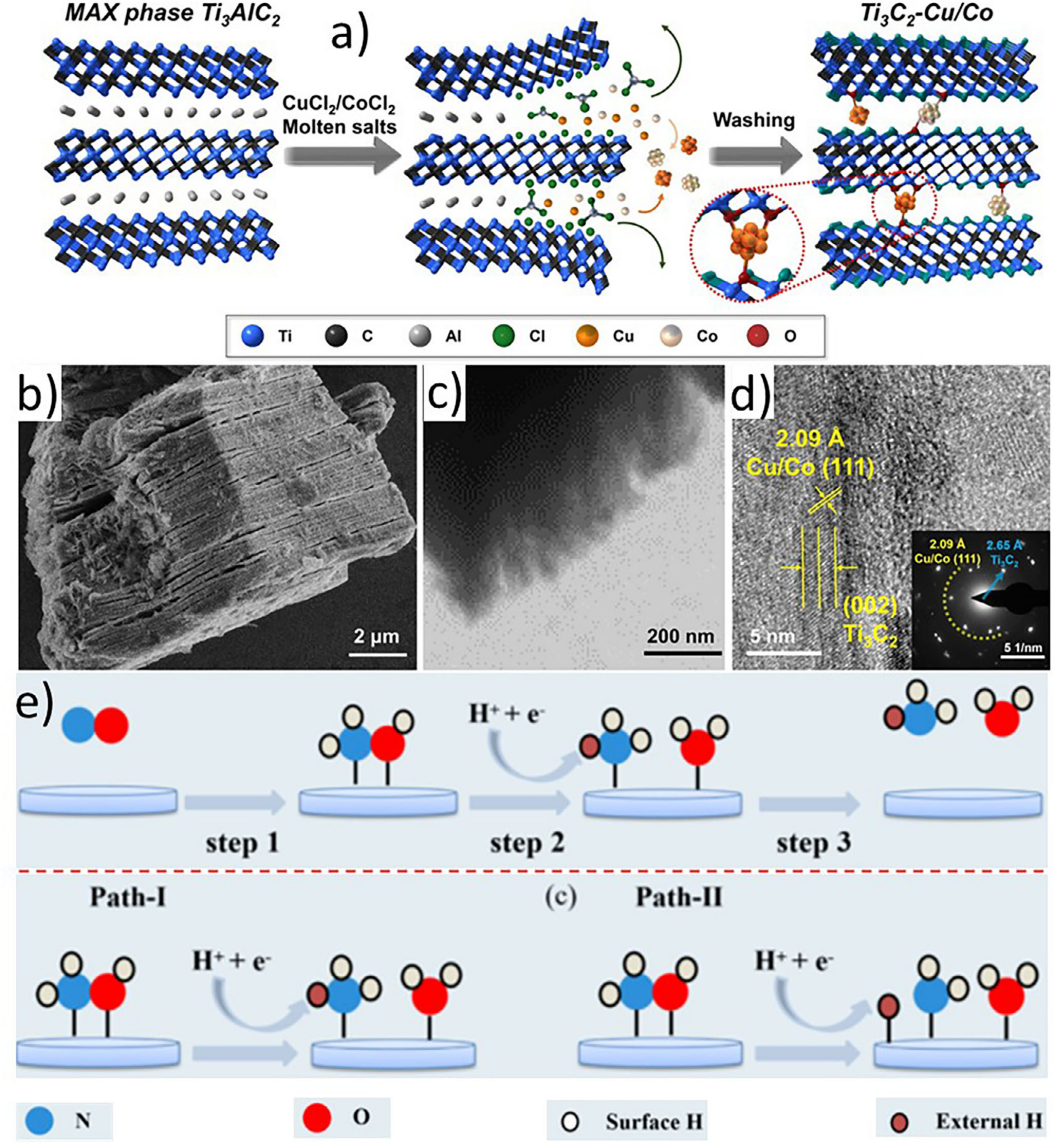
Morphological (a) Molten‐salt‐assisted transformation of Ti_3_AlC_2_ to Ti_3_C_2_ with in situ Cu incorporation. (b, c) Morphological reconstruction of the MXene sheets. (d) HRTEM evidencing crystalline Cu/Co domains interfaced with Ti_3_C_2_, reprinted with permission from Angew. Chem. (Wiley) [69]; (e) Comparative NO_x_‐to‐NH_3_ pathways via surface H*‐mediated H^+^/e^−^ coupling (Path‐I) and external hydrogen routes (Path‐II), illustrating interfacial hydrogenation control, reprinted with permission from ACS [70].

The dispersion and coordination environment of Cu species, together with the MXene surface morphology, fundamentally dictate catalytic behavior by modulating the electronic structure and interfacial reactivity. Figure 6a illustrates the molten‐salt‐assisted transformation of Ti_3_AlC_2_/MAX into Ti_3_C_2_/MXene sheets, followed by in situ anchoring of Cu/Co species and interfacial reconstruction [69]. The process enables uniform metal integration and strong electronic coupling with the MXene lattice after washing. Consistent with trends widely reported in nitrogen electroreduction, the structural evolution from densely stacked lamellae to hierarchical, fibrillar architectures markedly enhances interfacial transport dynamics. Such morphological reconstruction facilitates efficient electron conduction, ion diffusion, and NO_x_ mass transfer, thereby optimising triple‐phase boundary interactions at the catalytic interface. The stacked two‐dimensional morphology corresponds to the typical etched MXene structure and exhibits excellent in‐plane conductivity. However, as discussed in earlier MXene reports, excessive restacking restricts electrolyte penetration and limits the number of accessible edge sites, thereby hindering effective NO_x_ adsorption despite good electronic transport (Figure 6b). The introduction of finely dispersed Cu nanodomains on few‐layer MXene surfaces creates intimate metal‐support interfaces. The observed lattice fringes confirm the presence of crystalline Cu species strongly coupled to the conductive MXene scaffold. Such nanoscale decoration, widely emphasized in modern electrocatalysis literature, shortens electron‐transfer distances and promotes stabilisation of reaction intermediates, thereby improving selectivity and kinetics, as demonstrated by Figure 6c,d [69].

Two distinct hydrogenation pathways operate at the catalytic interface. Path‐I proceeds via surface‐bound hydrogen (H*) generated by H^+^/e^−^ coupling, enabling stepwise reduction via adsorbed NO_x_ intermediates as shown in Figure 6e. In contrast, Path‐II involves externally supplied hydrogen species, leading to an alternative intermediate configuration and altered protonation sequence. The dominance of the interfacial H*‐mediated route optimizes hydrogen utilization, lowers the kinetic barriers to the rate‐determining hydrogenation steps, and suppresses competitive HER. This pathway regulation strengthens NO_x_ binding while maintaining balanced H* coverage, thereby enhancing FE_(NH3)_ and steering selective NH_3_ formation [70].

**FIGURE 7:**
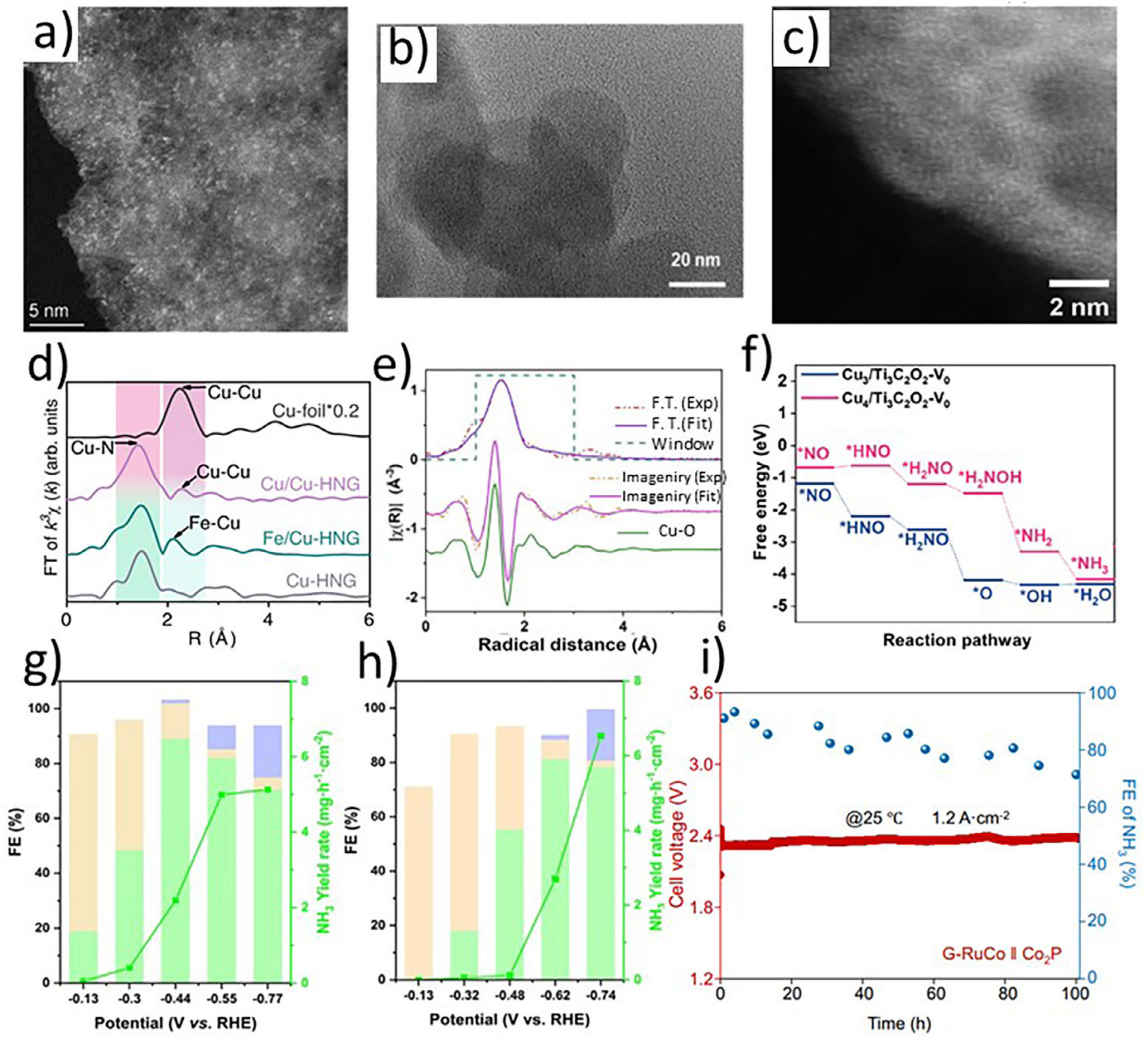
Cu single‐atom catalysts for NOx‐to‐NH_3_ electrocatalysis. (a–c) TEM and HAADF‐STEM images showing morphology and atomically dispersed Cu sites, reprinted with permission from Elsevier and Nature Mat [71, 72]. (d,e) Operando XAS/EXAFS confirming Cu‐N_4_ coordination and stable oxidation state, reprinted with permission from Nature Comm. and Nature Mater [72, 75]. (f) Reaction free energy profile for NO_x_ hydrogenation to NH_3,_ reprinted with permission from ACS Mat. Lett [73]. (g,h) NH_3_ yield and Faradaic efficiency vs potential, reprinted with permission from Elsevier [71]. (i) Long‐term stability over 24 h, reprinted with permission from Nature Comm [74].

The precise atomic dispersion and intrinsic stability of Cu sites on MXene are fundamental to achieving high‐efficiency NO_x_‐to‐NH_3_ electrocatalysis. TEM and HAADF‐STEM images (Figure 7a–c) reveal atomically isolated Cu atoms, and EDS mapping (Figure 7d) confirms uniform spatial distribution without aggregation, demonstrating atomic‐level structural control [71, 72]. Operando XAS/EXAFS spectra (Figure 7e) further reveal that Cu retains its N_4_ coordination and oxidation state under realistic reaction conditions, underscoring structural robustness [72]. This non‐clustered architecture maximizes the density of accessible active sites, promotes selective adsorption of NO_x_‐derived intermediates, and suppresses competing hydrogen evolution. Collectively, these features provide a mechanistic basis for stepwise hydrogenation, enhanced intermediate conversion, and highly selective NH_3_ formation, highlighting the importance of isolated Cu centers in guiding reaction pathways.

The reaction free energy profile (Figure 7f) shows energetically favorable NO_x_ hydrogenation pathways, while catalytic evaluation (Figure 7g,h) demonstrates that single‐atom Cu significantly enhances NH_3_ yield and FE [71, 73]. Long‐term electrolysis (Figure 7i) confirms sustained activity and operational durability over extended periods [74]. Together, these findings indicate that atomic‐level Cu control not only optimizes adsorption energetics and stabilizes reactive intermediates but also prevents aggregation and preserves catalytic integrity. The mechanistic advantage of non‐clustered Cu centers thus enables robust, selective, and efficient NO_x_‐to‐NH_3_ conversion, effectively bridging atomic‐scale structural precision with reaction kinetics and long‐term catalyst performance.

**FIGURE 8:**
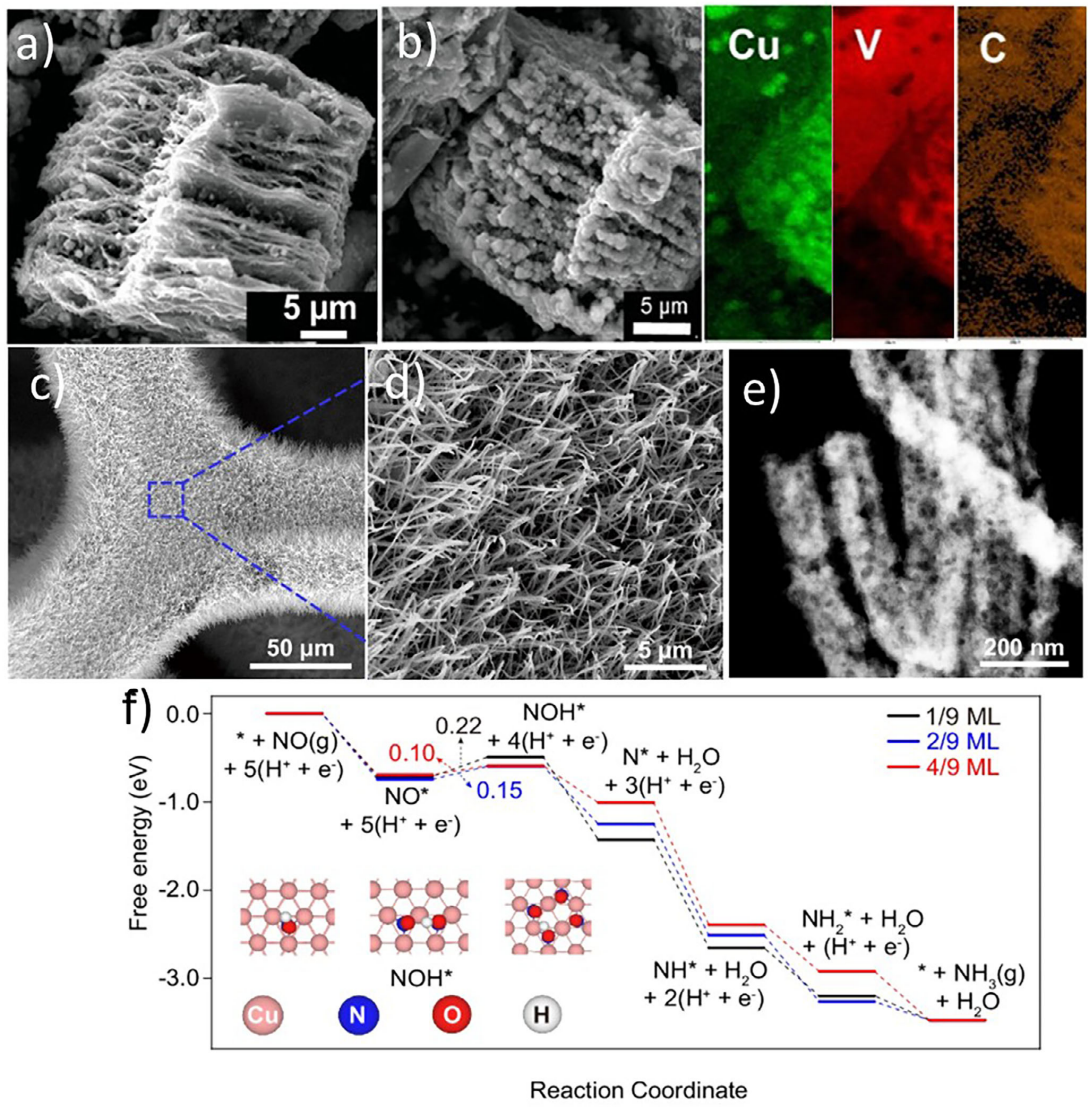
Morphological evolution (a,b) expanded and hierarchical Cu‐MXene structures with elemental mapping; reprinted with permission from ACS Nano [27]; (c) porous/foam‐like network; (d) nanofibrous architecture; and (e) nanoscale aggregates, illustrating multi‐scale structural features; reprinted with permission from Nature [29]. (f) Reaction pathway for the formation of NH_3_ on Cu(111) at different NO conversion FE for NH_3_ production, reprinted with permission from Nature [29].

Further catalytic efficiency improvements have been achieved by partially expanding the layered textures, which provide more exposed edges compared to fully compact sheets, yet the internal channels often remain irregular, as clearly seen in restacked or accordion‐like structures. Earlier comparative studies note that while wettability and ion access improve, performance gains can be limited when interlayer pathways are still tortuous. Further improvements have been carried out through hierarchical Cu ‐MXene architectures. The coexistence of micron‐scale backbones with dense nanoscale Cu features reflects a hierarchical design strategy frequently highlighted in high‐impact reports (Figure 8a,b). Such dual‐scale structuring increases active‐site density while preserving mechanical integrity and electrical continuity, offering a balanced platform for both activity and durability. The elemental mapping of (Cu‐V_2_C) showed a uniform spatial distribution of Cu over the MXene matrix, indicating genuine electronic interaction rather than simple physical mixing, a feature often associated with enhanced interfacial charge redistribution and long‐term operational stability [27].

It is widely reported that porosity plays a key role in catalytic activity, and open cellular morphologies significantly enlarge the contact area between the catalyst, the electrolyte, and the gaseous reactant. Similar porous electrodes in earlier gas‐phase electrocatalysis studies consistently demonstrate improved reactant diffusion and higher attainable current densities due to reduced mass‐transport limitations, as shown in Figure 8c. For further improvements, interwoven nanofibrous assemblies, such as one‐dimensional fibrous networks, maximize external surface exposure and establish continuous electron pathways. The literature on aerogel‐ and fiber‐based electrodes associates such structures with efficient bubble release and minimized local concentration gradients, which are beneficial for sustained reaction rates (Figure 8d). Similarly, Cu nanowires (with a diameter of 100–150 nm) provide abundant low‐coordination sites that are intrinsically active. However, modern studies caution that without strong support interaction, these domains may undergo coalescence or surface over‐binding during prolonged operation. The resulting monolithic porous Cu nanowire array, featuring micro‐, macro‐, and mesoporous channels (Figure 8e), provides multiscale transport regulation coupled with high‐density active sites. This structural synergy translates directly into optimized reaction energetics and enhanced NH_3_ selectivity, as evidenced by the lowered free‐energy barriers and superior FE presented in Figure 8f [27, 76].

### 3.2 Role of Coexisting Ions and Electrolyte Composition

**FIGURE 9:**
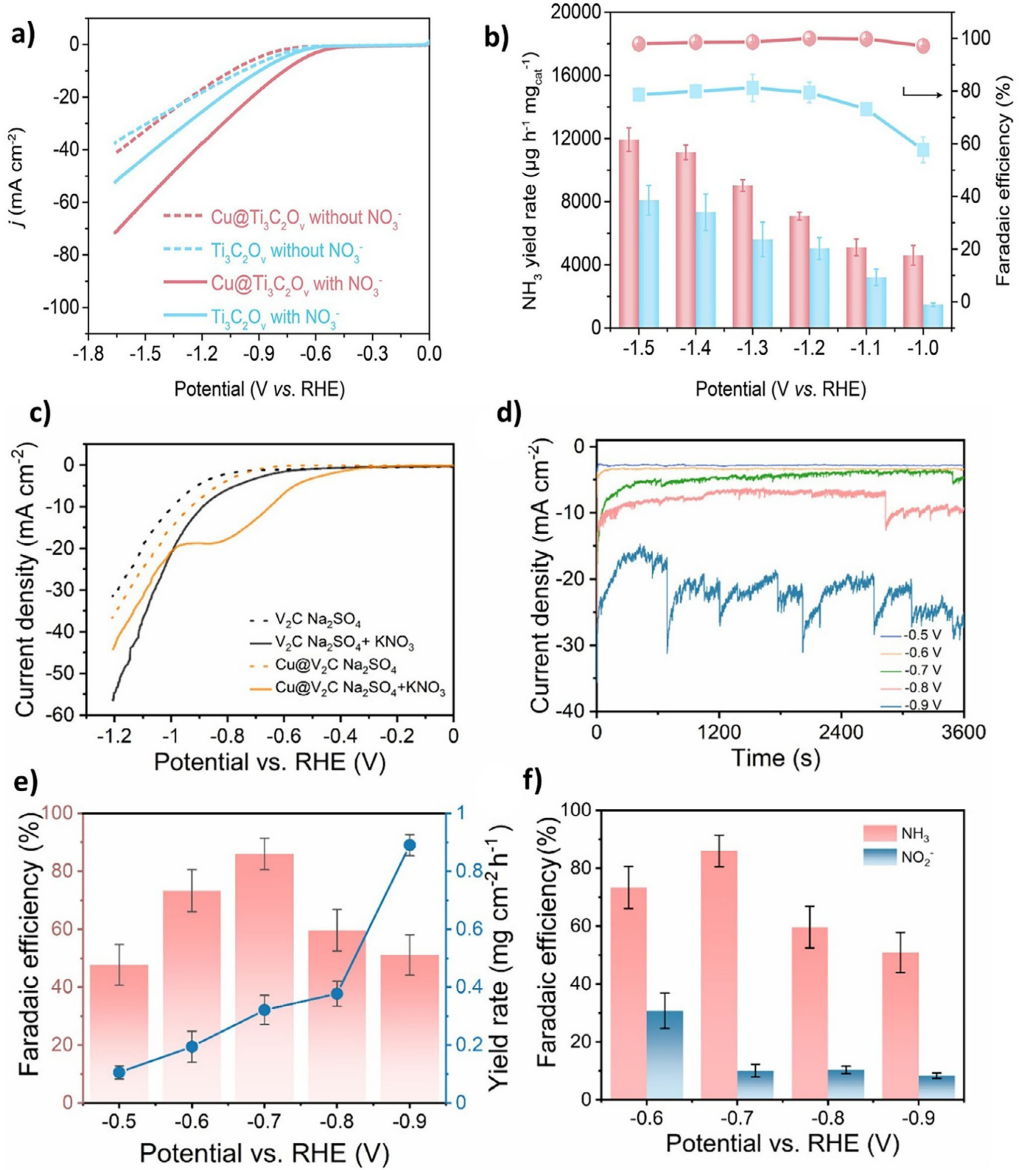
(a) LSV curves of Cu‐Ti_3_C_2_O_v_ and Ti_3_C_2_O_v_ in the absence (dotted lines) and in the presence of (solid lines); and (b) NH_3_ yield rates and FE% of Cu@Ti_3_C_2_O_v_ and Ti_3_C_2_O_v_ at −1.0 to −1.5 V vs. RHE, Electrochemical NH_3_ synthesis: (c) Linear sweep voltammetry of V_2_C and Cu@V_2_C in 0.5 m Na_2_SO_4_ (dashed line) and 0.5 m Na_2_SO_4_ +0.1 m KNO_3_ (solid line). (d) Chronoamperometry curves for Cu@V_2_C at different potentials. (e) NH_3_ yield and FE for Cu@V_2_C at different potentials. (f) FE for NH_3_ and NO_2_^−^ at different potentials, reprinted with permission from ACS Nano [27].

NO_x_ gas(s) are the key reactants for eNO_x_RR, and the components of the aqueous electrolyte have an equally important effect on catalytic performance. In particular, the coexisting cations and anions significantly alter the reaction microenvironment, adsorption behavior, and product selectivity [77]. Variations in ionic composition also influence the NH_3_ synthesis and unwanted by‐product generation, emphasizing the significance of electrolyte engineering and catalyst design, and anion impacts are the most important variables regulating eNO_x_RR activity. Strongly adsorbing anions such as SO_4_2^−^ or Cl^−^ can directly compete with NO_x_ species for active sites on the Cu‐MXene surface. This competitive adsorption inhibits crucial sites for NO and NO_2_ attachment, blocking the multi‐step hydrogenation process that leads to NH_3_ production [66]. Na_2_SO_4_ is a neutral electrolyte and commonly used to overcome restrictions by providing adequate ionic conductivity without strong interactions with catalytic surfaces. This Na_2_SO_4_ has established a benchmark medium for Cu‐MXene evaluations due to its inertness for adsorption and capacity to stabilize e^−^ transport without altering the reduction route. Tan et al. studied the performance of Cu‐incorporated oxygen‐vacancy‐rich MXene (Cu‐Ti_3_C_2_O_v_) [68]. In N_2_‐saturated electrolytes with 0.1 m NaNO_3_ and 0.1 m Na_2_SO_4_, the catalyst exhibited about 100% FE for NH_3_ during a wide potential range (−1.0 to −1.5 V vs. RHE) as given in Figure 9a,b.

Compared to pristine Ti_3_C_2_T_x_ and oxygen‐deficient Ti_3_C_2_O_v_, which obtained moderate NH_3_ selectivity (78‐88%), but created significant NO_2_ by‐products. At the same time, Cu‐Ti_3_C_2_O_v_ demonstrated improved current densities, greater NH_3_ yield rates, and robust suppression of NO_2_^−^ generation. DFT calculations revealed that Cu@Ti_3_C_2_O_v_ binds strongly to NO_3_^−^ and *NO_2_ intermediates, resulting in a high‐energy barrier to NO_2_^−^ release. This combination stabilized the reduction pathway to NH_3_, demonstrating the synergistic importance of electrolyte composition and MXene structural engineering for achieving high efficiency. Nittoor‐Veedu et al. report a high‐performance Cu‐incorporated V_2_C catalyst prepared through a molten‐salt route involving selective CuCl_2_‐assisted etching [27]. Linear sweep voltammetry (LSV) was carried out using the Cu‐V_2_C as the catalyst in 0.5 m Na_2_SO_4_ and 0.1 m KNO_3_ + 0.5 m Na_2_SO_4_ to study their performance toward NO_3_RR. To enable a direct comparison, identical measurements were performed on the V_2_C catalyst without Cu, as shown in Figure 9c. The lower reduction potential of Cu@V_2_C and its greater shift in reduction potential observed with and without nitrate ions indicate that Cu@V_2_C exhibits intrinsic electrochemical activity toward NO_3_RR under these electrolytes. The H‐cell setup helps to reduce the back oxidation at the anode, leading to improved catalyst efficiency. During electrolysis, a higher current density is observed with Cu‐V_2_C (Figure 9d) compared to pure V_2_C, indicating that Cu insertion enhances electrochemical performance [27].

After electrolysis, the catholyte collected from the H‐cell was analyzed for NH_3_ production using a colorimetric method combined with UV–vis spectroscopy. With increasing cathodic potential, the FE (Figure 9e) initially increased from ∼50% at −0.5 V to a maximum of ∼83% at −0.7 V vs RHE, then declined at more negative potentials. In contrast, the NH_3_ yield continuously increased, reaching ∼900 cm^−2^ h^−1^ at higher overpotentials. The superior performance at −0.7 V vs RHE highlights an optimal balance between NO_x_ activation and suppression of competing reactions, arising from the synergistic interaction between the V_2_C MXene matrix and interlayer‐infused Cu species. Meanwhile, the FE for NO_2_ remained below 30% and decreased with increasing potential, confirming the high selectivity of Cu@V_2_C toward NH_3_ production (Figure 9f). Based on the maximum FE, −0.7 V vs. RHE was selected for stability evaluation, where the catalyst maintained ∼83% FE with an NH_3_ yield of 320 µg cm^−2^ h^−1^. Extending this Cu‐infusion approach for Ti_3_C_2_/MXene further verified its generality, yielding ∼70% FE and an NH_3_ production rate of ∼450 µg cm^−2^ h^−1^ at −0.6 V vs. RHE [78]. These results indicate that Cu incorporation effectively controls the MXene electronic structure and reaction microenvironment by offering a broadly applicable platform with high‐performance NO_x_‐to‐NH_3_ electrocatalysis.

Cations are generally neglected, yet they play an important role in determining interfacial behavior during eNO_x_RR. Monovalent alkali ions (K^+^, Na^+^, and Cs^+^) can adjust the electric double layer at the electrode‐electrolyte interface, alter the surface charge distribution, and reduce HER by reducing H^+^ movement to the catalytic surface [67]. The addition of K^+^ has been established to boost e^−^ density near metal active sites, assisting in faster NO_x_ adsorption and desorption, enhancing the reduction kinetics, and promoting NH_3_ selectivity. But divalent cations such as Ca^2^^+^ and Mg^2^^+^have a negative influence and decrease the efficiency. Their capacity to produce surface precipitates or cause scaling reduces accessibility to active sites and disrupts the delicate stability of the catalyst‐electrolyte interface, resulting in a decline in efficiency. The effects of coexisting ions are amplified in Cu‐MXene systems due to MXene's stacked structure and surface chemistry. Studies on Fe_1_Cu_2_‐MXene indicated that Na_2_SO_4_ provides a stable electrolyte environment, allowing constant catalytic activity. Large quantities of oxidising ions, such as Fe^2+^/Fe^3+^, corroded the MXene substrate and inactivated the anchoring active sites. XPS studies revealed that Cu species were mostly present as Cu‐Cl and Fe as FeOOH. Unexpectedly, these species were stabilized via electrostatic interactions with negatively charged MXene layers, forming a tandem configuration that boosts charge transfer, lowers HER overpotential, and improves long‐term utilization across multiple cycles. As a result, these findings verify the relevance of both anions and cations in creating the catalytic environment of Cu‐MXene‐based systems [79]. Neutral electrolytes, such as Na_2_SO_4_, provide a favorable environment for effective NO_x_ reduction, but strongly adsorbing anions compromise selectivity by competing with NO_x_ for surface sites. Similarly, monovalent cations can help reduce NO_x_ by stabilizing surface charge distributions and hindering HER, but divalent ions often cause surface fouling. The example studies of Cu‐Ti_3_C_2_O_v_ and Fe_1_Cu_2_‐MXene demonstrated that the electrolyte properties interact synergistically with the structural and electrical properties of Cu‐MXene catalysts.

Electrolyte engineering for catalyst development must be dealt with as a strategic design parameter. Stabilizing active sites, minimizing parasitic reactions, and improving the NH_3_ selectivity can be achieved by carefully selecting supportive electrolytes, optimizing cation/anion concentrations, and minimizing detrimental ion degradation. Therefore, electrolyte engineering and sophisticated Cu‐MXene catalysts represent a promising approach to developing high‐performance, long‐lasting, and scalable eNO_x_RR systems.

### 3.3 Effect of pH on NO_x_ Gas Reduction

The pH of the electrolyte plays a key role in regulating interfacial chemistry, adsorption kinetics, and the progress of competing reactions during the electrochemical conversion of NO_x_ to NH_3_. Unlike ionic N‐compounds like NO_3_^−^ or NO_2_^−^, the dissolution, diffusion, and surface adsorption of gaseous NO and NO_2_ are strongly affected by H^+^ concentration in the electrolyte, and the resulting pH‐dependent interactions at the catalyst electrolyte boundary [80]. These interactions determine whether active sites are available for NO_x_ adsorption or occupied by protons or hydroxide ions, and have a direct influence on the selectivity, FE, and overall performance for the eNO_x_RR.

In strongly acidic electrolytes with high H^+^ concentrations, HER becomes exceptionally competitive. The H^+^ are easily adsorbed onto active catalytic sites, where they deplete e^−^ to produce H_2_ [63]. This competitive (H^+^) adsorption reduces NO_x_ adsorption and prevents the multi‐step hydrogenation pathways that produce NH_3_. Thus, the FE_NH3_ declines dramatically, and more current is diverted toward HER. At the same time, H_2_ production is not undesired in several energy applications; it represents a parasitic reaction in the context of eNO_x_RR. This decreases the efficiency of NH_3_ synthesis and compromises process selectivity. Cu‐MXene systems have demonstrated remarkable selectivity for NH_3_ in slightly acidic circumstances. Cu‐MXene catalysts maintained steady NO_x_ conversion efficiencies at pH = 3 and achieved nearly 98.8% NH_3_ selectivity [19]. This performance indicates that, at carefully controlled acidity levels, proton availability can expedite the hydrogenation stages of *NO and *ONH intermediates. However, this advantage is only realized when the catalyst surface is designed to reduce HER competition, as in Cu‐MXene, where strong conductivity and tunable surface chemistry aid the stabilization of nitrogen‐containing intermediates. Neutral to mildly alkaline conditions (pH = 6–8) have been identified as optimal for balancing NO_x_ reduction with reduced HER. At these pH levels, the H^+^ concentration is adequate to allow hydrogenation processes, but not high enough to saturate the catalyst surface with available H^+^. This equilibrium keeps active sites accessible for NO and NO_2_ adsorption, while creating an interfacial environment that promotes stepwise hydrogenation of intermediates into NH_3_. Thus, the Cu‐MXene catalysts produce NH_3_ more efficiently and selectively under these circumstances. The enhanced performance is ascribed to lower HER competition enabled by more stable intermediate adsorption and favorable interfacial charge distributions. A neutral to slightly alkaline pH is optimal for eNO_x_RR forward reaction pathways, resulting in high NH_3_ production while minimising by‐products such as N_2_O or NO_2_^−^. At highly alkaline pH levels (around 12 or higher), the situation changes significantly. Although NO_x_ conversion remains reasonably high (∼82%), NH_3_ selectivity drops dramatically due to the above‐mentioned reasons [19, 81]. The reduced H^+^ concentration slows the hydrogenation steps required to convert adsorbed intermediates into NH_3_. Excess hydroxide ions (OH^−^) in the electrolyte compete aggressively with NO_x_ species for adsorption on the catalyst surface. By occupying active sites, OH^−^ prevents NO and NO_2_ molecules from adsorbing, thereby preventing the generation of reactive intermediates including *NO_2_, *ONH, and *NH_2_. In addition, high OH^−^ adsorption can change the catalyst's electronic structure, affecting the adsorption of N‐species. These modifications reduce selectivity for NH_3_ and increase the chance of undesired by‐products. Excessive OH^−^ surface coverage has been linked to decreased current densities and reduced long‐term performance. Although alkaline electrolytes can reduce the HER, they have limits such as enlarged OH^−^ competition and slower hydrogenation kinetics [82].

Cu‐MXene catalysts validate the precise balancing of pH effects in eNO_x_RR. Their surface terminations, conductivity, and tunability make them less susceptible to HER than many traditional catalysts, though they remain heavily pH‐dependent. In alkaline environments (pH ≈ 12), selectivity decreases to ∼78.9% due to increased HER and altered adsorption circumstances [19, 33]. These findings highlighted that Cu‐MXene catalysts perform best in acidic‐to‐neutral conditions, where H^+^ availability promotes efficient hydrogenation and prevents HER domination. To ensure consistent performance throughout long electrochemical processes, it is necessary to stabilize the electrolyte pH. Variations in pH during operation can affect catalytic activity, FE, and selectivity. Buffering systems, such as phosphate‐buffered saline (PBS), have been used to control pH and maintain an ideal interfacial environment. Buffered electrolytes help maintain stable adsorption and hydrogenation pathways by minimising rapid changes in H^+^ or OH^−^ concentration. This improves both short‐term efficiency and long‐term catalyst stability.

The impact of pH on eNO_x_RR demonstrated significant optimization in an electrolyte environment for selective NH_3_ production and the overall system efficiency. Cu‐MXene catalysts benefit from pH values in the acidic‐to‐neutral range for efficient NO_x_ adsorption, intermediate stabilisation, and stepwise hydrogenation. Highly acidic conditions improve HER, whereas highly alkaline ones cause OH^−^ competition. To improve process efficacy, future research should look into dynamic pH control schemes, improved buffering systems, and operando interfacial chemistry surveillance techniques. Integrating electrolyte engineering with Cu‐MXene's structural and electrical advantages can yield efficient and long‐lasting devices for NH_3_ production from gaseous NO_x_.

### 3.4 Influence of Applied Potential

The applied potential is one of the most important external factors that alter both thermodynamic feasibility and kinetic advancement in the eNO_x_RR process. Tuning the electrode potential can regulate e^−^ transfer rates, NO_x_ adsorption, and activation, as well as competition between desired NH_3_ production and parasitic side reactions like HER [66]. A well‐defined potential window not only controls the reaction progression but also significantly influences the pathway's selectivity and catalyst stability over time. Empirical and theoretical studies show that a slightly negative potential window of −0.5 to −0.9 V vs. the RHE is ideal for efficient NO_x_ conversion to NH_3_. In this regime, the driving force is sufficient to boost e^−^ transfer to adsorbed NO and NO_2_ intermediates, thereby improving their successive hydrogenation to NH_3_, while also promoting additional HER. This balance optimize FE and product selectivity, ensuring that the majority of e^−^ are sent to the desired NH_3_ pathway. Catalysts in this potential window stabilize the adsorption geometry of intermediates, suppressing competing by‐products (HER/N_2_O). This occurs when N‐species undergo unwanted N–N coupling. However, operating outside of this optimal timeframe creates substantial issues. At highly negative potentials, the initial rate of NO_x_ conversion may rise due to increased e^−^ availability, but HER becomes more prominent over time. This competing reaction not only diverts e^−^ away from NO_x_ reduction but also obstructs catalytically active sites with adsorbed hydrogen (*H). The presence of these sites disrupts the delicate multi‐step hydrogenation sequence required for efficient NH_3_ synthesis, resulting in decreased FE and overall NH_3_ yield. Potentials close to zero or slightly positive relative to RHE are insufficient to adsorb or activate NO_x_ molecules efficiently. In these conditions, intermediates are unstable, e^−^ transportation slows, and NH_3_ production is minimal.

Maintaining meticulous control over the applied potential is not just a matter of experimentation; it is intricately connected to catalyst design. Catalysts with improved conductivity, optimized surface finishes, and favorable active‐site configurations can greatly reduce the intrinsic overpotential required to initiate NO_x_ reduction. Cu‐MXene systems, in particular, have established distinct advantages, including a wide potential window, while maintaining good NH_3_ selectivity. Cu nanoparticles or atomically distributed Cu sites on highly conductive MXene substrates boost charge‐transfer kinetics, while tailoring the interface's electrical structure. This synergy decreases the energy barriers related to critical chemical intermediates, enabling efficient NH_3_ generation at lower energy. Cu‐MXene catalysts stabilize the *NO_2_ intermediate, a key bottleneck in the reduction pathways. Cu‐MXene surfaces hinder NO_2_^−^ accumulation, resulting in a smooth hydrogenation cascade to NH_3_ production. This maintenance minimizes the RISK of side reactions, such as partial reduction products or intermediate re‐oxidation, thus increasing both selectivity and efficiency. Several reports have highlighted the effect of voltage on Cu‐based electrocatalysts. Wan et al. confirmed that Cu catalysts outperform other transition metals for NH_3_ production within a narrow potential window of 0 to −0.3 V vs. RHE [83]. Although *H adsorption hindered the overall current density in this regime, selective NO reduction to NH_3_ remained dominant, demonstrating Cu's favorable catalytic properties. In contrast, at potentials more positive than 0.3 V vs. RHE, the risk of unwanted N‐N coupling arises, supporting N_2_O as a competitive byproduct.

**FIGURE 10:**
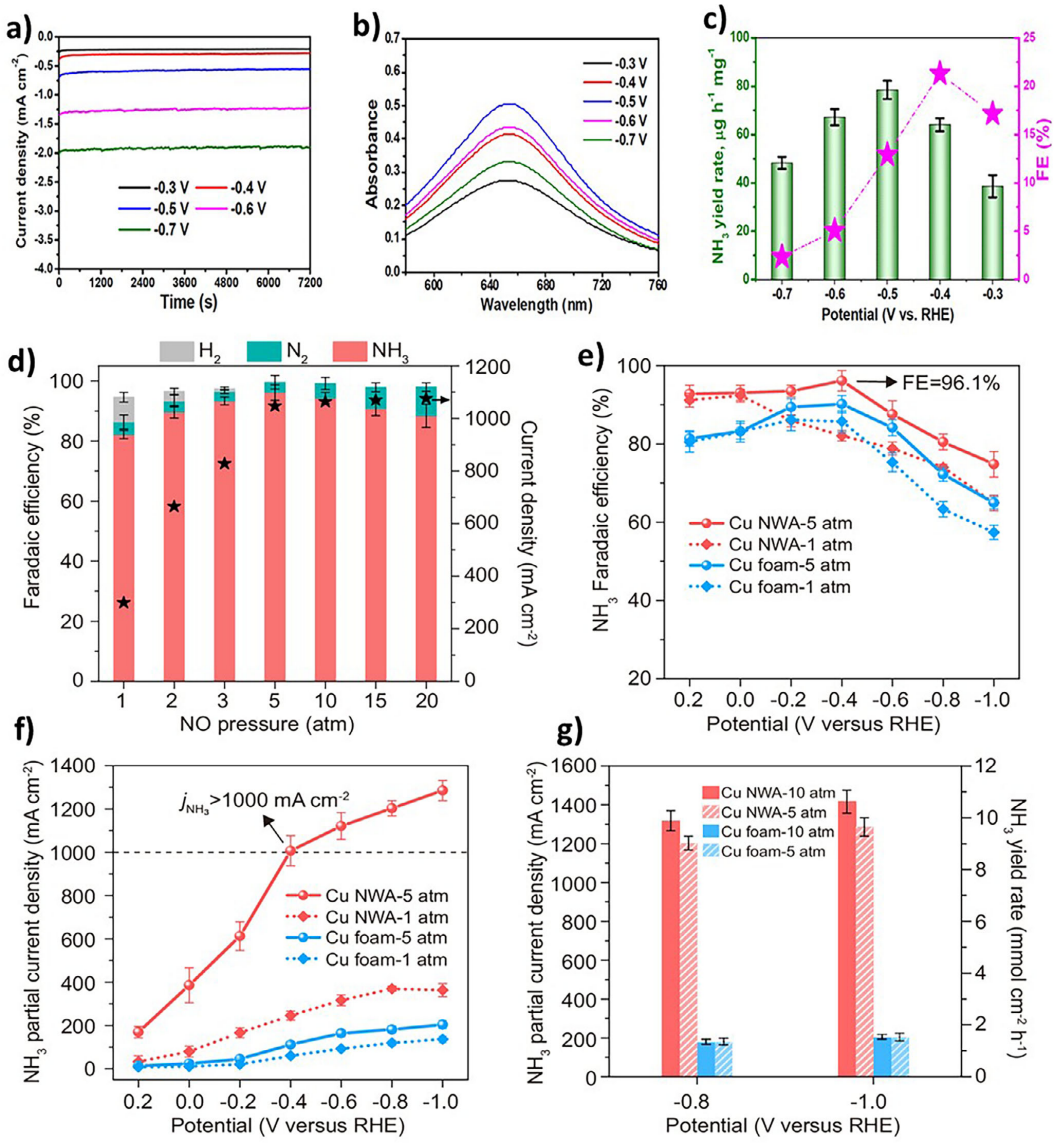
(a) MQDs/Cu chronoamperometry curves at different potentials during 2 h of NORR electrocatalysis, together with matching (b) Absorption spectra of UV–vis and the consequent (c) NH_3_ yields and FE's, reprinted with permission from Energy & Environ. Mat. (Wiley) [20], (d) Effect of NO pressure on product distribution and total current density during NORR over Cu NWA at − 0.4 V vs. RHE at 25°C. NH_3_ FE (e) and NH_3_ current density (f) over Cu NWA and commercial Cu foam at different applied potentials at 25 °C under 1 atm, 5 atm NO, respectively. (g) The NH_3_ partial current densities and corresponding NH_3_ yield rate of Cu NWA and Cu foam under 5 and 10 atm NO at 25°C, respectively, reprinted with permission from Nature Com [29].

MXene‐supported catalysts exhibit comparable sensitivity. Ti_3_C_2_O_v_ had FE_NH3_ values of 78.5%–88.4% between −1.1 and −1.5 V vs. RHE, beating pristine Ti_3_C_2_T_x_ MXene in the same range, as shown in Figure 1b. The Ti_3_C_2_T_v_ catalyst obtained a maximum NH_3_ production rate of 8098.5 µg h^−^^1^ mgcat^−^^1^ at −1.5 V, while delivering greater selectivity than the virgin MXene. At −1.0 V, Ti_3_C_2_O_v_ had a FE_NH3_ of 52.8%, which was much higher than Ti_3_C_2_T_x_ (36.8%). Cu‐modified Ti_3_C_2_O_v_ improved performance by stabilising the *NO_2_ intermediate and inhibiting NO_2_^−^ by‐product production. This resulted in higher NH_3_ selectivity across the examined potential window. Wang et al. [84]. further developed a Cu‐V_2_C MXene catalyst via atomic layer deposition. The optimized 15Cu‐V_2_C material obtained a remarkable NH_3_ yield rate of 11.25 mol gcat^−^^1^ h^−^^1^ with near‐unity FE at −1.0 V vs. RHE and maintained outstanding stability over 30 cycles. Carefully engineered Cu‐MXene hybrids not only move the onset potential to more favorable values, but also expand the possible window for high‐performance NH_3_ generation. Density functional theory (DFT) simulations supplement these experimental findings by highlighting how the applied potential affects adsorption energetics. Cu‐Ti_3_C_2_O_v_ shows significant binding to NO_3_^−^ and *NO_2_ intermediates, with the production of NO_2_^−^ requiring a high energy barrier. This explains the catalyst's ability to suppress NO_2_^−^ release and channel the pathway selectively toward NH_3_ formation. Importantly, these models show that extremely negative potentials reduce the energy barriers to HER, shifting the competitive balance away from NO_x_ reduction. The interaction between the applied potential and surface energetics dictates whether electrons are steered toward NH_3_ production or toward parasitic side reactions. Similarly, in another study, Cheng et al. integrate porous Cu nanosheets with Ti_3_C_2_T_x_‐MXene quantum dots (MQDs) to create a unique heterostructure, MQDs/Cu as a long‐lasting and efficient NORR catalyst [20]. A combination of the spectrophotometric method and the chronoamperometry test (Figure 10a) is used to evaluate the NORR performance of MQDs/Cu. (Figure 10b) [32]. Figure 10c shows the greatest NH_3_ output of 78.5 µg h^−1^ mg^−1^ at ‐0.5 V, and the highest FE of 21.3% at −0.4 V, indicating that the characteristic NORR activity's volcano‐shaped dependence on applied potential is comparable to or superior to that of the majority of previously documented catalysts. The rise in HER at high potentials is the reason why the NORR activity of MQDs/Cu is significantly decreased beyond the potential of −0.5 V [85, 86]. Remarkably, MQDs/Cu demonstrated a synergistically improved NORR activity that was far better than that of pure MQDs and Cu, surpassing most of the most advanced NORR catalysts available. To ensure ENRA's excellent performance, potentials with values between −0.3 and −0.8 vs RHE are chosen [87]. Instead of −0.8 V vs. RHE, the highest NO_3_ conversion efficiency (93.1%) and FENH_3_ (86.5%) are observed at −0.7 V. Studies from the literature suggest that an excessive HER side reaction is responsible for this result [88]. Therefore, the operation voltage for the next batch experiment has been set at −0.7 V. Additionally, Cu_x_O/Ti_3_C_2_T_x_ showed significant electrocatalytic performance with a FE of 48% at −0.7 V vs. RHE and NH_3_ output of 41 982 µg h^−1^ mcat^−1^ [39].

The electrocatalytic NO reduction reaction (NORR) represents a compelling strategy for sustainable NH_3_ production while simultaneously mitigating NO pollution. Nevertheless, achieving both high NH_3_ productivity and prolonged operational stability remains a major barrier to industrial implementation. Yang et al. established an in situ constructed hierarchical porous Cu nanowire array monolithic electrode integrated into a pressurized electrolyzer, allowing precise control of eNORR kinetics and thermodynamics. This system offers an industrially relevant NH_3_ partial current density of 1007 mA cm^−2^ with an exceptional FE of 96.1%, while maintaining a stable process at 1000 mA cm^−2^ for 100 h [29]. The electrocatalytic efficiency of Cu NWA monolithic electrode toward eNORR was evaluated using an H‐type pressurized electrolyzer under varying NO partial pressures (*pNO*). The pressure‐sensitive eNORR activity was first examined on the Cu NWA catalyst. At a fixed cathodic potential of −0.4 V vs. RHE, the current density increased markedly from 299 to 1047.4 mA cm^−2^ as *pNO* rose from 1 to 5 atm, approaching a plateau at elevated pressures (Figure 10d). This pronounced improvement in current density at elevated pressures is attributed to improved NO dissolution and faster mass transport within the electrolyte, which collectively promote the NORR process. In addition, the NO partial pressure applies a pronounced influence on the FE_NH3_. As the NO pressure rises from 1 to 5 atm, FE increases steadily from 82.1% to 96.1% (Figure 10d). This improvement is primarily attributed to increased NO surface coverage, which effectively avoids the HER. Relative to operational 1 atm, the Cu NWA electrode consistently brings substantially higher total and NH_3_ partial current densities at 5 atm across the entire potential range studied (Figure 10e). Notably, upon further increasing the NO partial pressure to 10 atm, the NH_3_ partial current density reaches as high as 1415.4 mA cm^−2^ at −1.0 V vs. RHE. Under pressurized conditions, the HER is effectively suppressed over a broad potential window, thereby favoring the eNORR toward NH_3_ formation. These results clearly demonstrate the beneficial effect of elevated NO pressure on eNO_x_RR activity and selectivity. Moreover, at 1 atm NO, the Cu NWA electrode consistently outperforms Cu foam, showing higher NH_3_ partial current densities at all applied potentials (Figure 10f). These findings suggest that the Cu NWA electrode delivers a substantially higher density of active sites for eNORR‐driven NH_3_ production. In contrast, Cu foam exhibits only a marginal increase in NH_3_ partial current density at 5 atm, likely due to its limited accessible catalytic active sites. At an NH_3_ partial current density of 1415.3 mA cm^−2^, an exceptional NH_3_ production rate of 10.5 mmol cm^−2^ h^−1^ is achieved at −1.0 V vs. RHE under 10 atm NO, beyond that of commercial Cu foam operated at 1 atm by more than an order of magnitude, and surpassing previously reported eNORR systems by almost two orders of magnitude (Figure 10g). The combination of the active‐site‐rich Cu NWA construction and elevated NO pressure, which improves mass transport, effectively modulates both the kinetics and thermodynamics of the eNORR [19, 68]. This synergistic effect markedly improves the electrochemical conversion of NO to NH_3_, resulting in an exceptionally high NH_3_ partial current density.

Beyond influencing efficiency and selectivity, applied potential directly affects catalyst stability. Operating at appropriate potentials reduces the possibility of surface deactivation by intermediates, while minimising mechanical and structural stress arising from tedious redox cycles. Cu‐MXene catalysts exhibit longer operational lifetimes and deliver constant NH_3_ yields over extended reaction times. Continuous process at severe negative potentials, on the other hand, encourages catalyst deterioration by boosting hydrogen bubble formation, surface rearrangement, and, eventually, active‐site loss. These findings reveal that Cu‐MXene catalysts require an optimized potential window to balance e^−^/H^+^ transfer, inhibit HER, and maintain high NH_3_ selectivity. Combining conductive MXene scaffolds with catalytically active Cu sites reduces inherent overpotential, improves intermediate stability, and expands the operational range for efficient NH_3_ generation. This dual advantage not only lowers energy consumption but also increases catalyst longevity and scalability. Future research should focus on combining potential optimisation with real‐time operando surveillance to provide an adaptive voltage regulator in dynamic electrochemical conditions. Such adaptations could improve system resilience, allowing Cu‐MXene electrocatalysts to play a crucial role in sustainable, scalable electrochemical NH_3_ synthesis from NO_x_.

### 3.5 Effect of NO_x_ Gas Concentration

The concentration of NO_x_ species introduced into the electrochemical system is one of the most important elements influencing the kinetics, selectivity, and overall performance of the eNO_x_RR procedure. NO_x_ molecules dissolve in the electrolyte, diffuse to the catalyst surface, adsorb at active sites, and reduce to NH_3_. The bulk concentration of NO_x_ significantly influences each of these phases by affecting both mass‐transport dynamics and surface coverage of reactive intermediates. As a result, optimising NO_x_ concentration is crucial to balance the catalytic efficiency, reduce unwanted side reactions, and ensure long‐term catalyst durability. At low NO_x_ concentrations, the reaction is susceptible to mass‐transport limitations. When there are insufficient reactant molecules in the electrolyte, the movement of NO_x_ species to the catalyst surface is delayed. This leads to a decrease in surface coverage of crucial intermediates (*NO_3_, *NO_2_, *NO), which are obligatory for beginning the hydrogenation cascade and eventually NH_3_ production. The catalyst's active sites are neglected, resulting in reduced NH_3_ yield and FE%. In these situations, the reaction atmosphere cannot sustain continuous e^−^/H^+^ transfer cycles, resulting in slow kinetics and incomplete transformation. According to studies, sophisticated delivery approaches, such as gas recirculation, pressurized operation, or membrane‐based pre‐concentration, are essential to circumvent these bottlenecks by increasing the flux of NO_x_ molecules to the catalyst‐electrolyte [66]. These approaches ensure that even dilute NO_x_ streams, such as those from ambient pollutants, can be efficiently transformed into usable NH_3_ at reasonable reaction rates.

In contrast, maintaining a suitable concentration window permits efficient surface coverage without overloading the catalyst. In this regime, NO_x_ molecules adsorb homogeneously across the catalyst surface, resulting in a balanced reaction atmosphere, and the intermediates undergo stepwise hydrogenation. This removes site‐hindering, stabilizes the electrical structure of active sites, and reduces byproduct production. Sufficient but not excessive NO_x_ coverage preserves adsorption‐desorption equilibrium, allowing newly generated NH_3_ molecules to desorb easily without saturating the surface. Catalysts can attain maximal NH_3_ selectivity and high FE under balanced conditions, leading to increased productivity and longer catalytic lifetimes. Several studies have shown a favorable relationship between mild NO_x_ partial pressures and higher NH_3_ production rates, provided the system is operated within this concentration range [29, 66]. On the other hand, very high NO_x_ concentrations pose a unique challenge. While initially enhancing reactant availability, excess NO_x_ molecules might assemble at the catalyst surface, saturating adsorption sites and limiting the desorption of NH_3_ products. Oversaturation causes surface deactivation, where excess *NO_2_ and related intermediates block active sites and impede further hydrogenation. Under these conditions, NH_3_ output declines and undesirable by‐products, such as NO_2_^−^ and N_2_O, are formed. Long‐term exposure to high NO_x_ concentrations can also compromise catalyst durability, affect surface shape and electrical characteristics, and reduce electrode reusability.

The effect of concentration on catalytic efficiency has been convincingly confirmed in Cu‐MXene systems. For example, Fe_1_Cu_2_@MXene showed virtually full nitrate conversion and NH_3_ selectivity of 95%–99% at moderate NO_3_^−^ concentrations (∼100 mg L^−^^1^) [19]. However, at lower concentrations, the process became diffusion‐limited, leading to poorer NH_3_ harvests and efficiency. In contrast, at extremely high concentrations, the accumulation of NO_2_^−^ intermediates partially poisoned the active sites, reducing selectivity. These findings verify that Cu‐MXene catalysts, despite their high conductivity and strong intermediate stabilisation capacity, are vulnerable to concentration‐dependent issues and thus require optimized feed management. Likewise, Tan et al. explored the influence of NO_3_^−^ concentration on the efficiency of Cu‐Ti_3_C_2_O_v_ catalysts [68]. Their results showed a strong correlation between concentration and NH_3_ harvest, particularly at an applied potential of −1.2 V vs. RHE. Yields were limited below 100 mM due to insufficient surface coverage, while concentrations above this threshold resulted in oversaturation and reduced accessibility of free active sites. At 100 mm, NH_3_ selectivity was approximately 100%, indicating total nitrate utilization during the reaction. The selectivity trends for NO_2_^−^ (S_NO2_^−^) and NH_3_ (S_NH3_) revealed that intermediate NO_2_^−^ concentrations deteriorated linearly with time as NH_3_ selectivity increased, indicating effective conversion by consecutive hydrogenation. However, when the concentration surpassed acceptable levels, NH_3_ desorption became slow, highlighting the significance of careful feed management.

These experimental and computational evaluations highlight the dual challenge posed by NO_x_ concentration: too little, and the process becomes kinetically imperfect; too much, and the catalyst suffers from overloaded, site‐blocking, and long‐term degradation. Addressing this balance requires both a basic understanding and practical engineering acknowledgment. At the laboratory scale, feed concentration can be finely adjusted to increase FE% and yield rates. At the industrial scale, however, additional approaches are necessary to handle fluctuating or dilute NO_x_ streams from real discharge sources. Approaches such as precise dilution of high‐NO_x_ gases or pre‐concentration of low‐concentration exhausts offered a route to stabilize reactant supply. Gas‐handling techniques, including recirculation systems and membrane separation, are also gaining attention as scalable solutions to maintain the best concentration windows during continuous processes [66]. In conclusion, optimizing NO_x_ concentration is essential for achieving high NH_3_ yields, selectivity, and catalyst stability in Cu‐MXene systems. By maintaining a concentration series that balances enough surface coverage with efficient product desorption, the electrocatalytic environment can be fine‐tuned to defeat side reactions, prevent catalyst deactivation, and improve overall performance. Future work should focus on linking catalyst innovations with progressive feed delivery policies, enabling Cu‐MXene‐based eNO_x_RR technologies to transition from laboratory confirmation to scalable, industry‐relevant solutions.

### 3.6 Comparison of Cu@MXene with Other Catalysts

Compared to other traditional catalysts for NO_x_ reduction, Cu‐based MXenes offer different advantages in terms of activity, selectivity, and working stability. Commonly used noble metal catalysts Ru, Au, and Pt demonstrate high intrinsic activity but are expensive, with limited stability and vulnerability to poisoning under reaction conditions. Pure Cu catalysts, while cost‐effective, often display limited stability due to accumulation or oxidation, especially under long‐term electrochemical operation. The comparison of the stability, selectivity, and activity of Cu‐based MXenes with those of other catalyst systems is presented in Table 2.

**TABLE 2:** General Comparative Table: Cu‐Based MXenes vs. Other eNO_x_RR Catalysts [30, 67, 89]

Catalyst	NH_3_ Yield (µg mg^−^^1^ h^−^^1^)	Faradaic Efficiency (%)	Potential V vs. RHE	Stability	Cost	References
Cu‐Based MXenes (e.g., Cu@Ti_3_C_2_T_x_)	High (100‐300)	Moderate (40–65)	—	Good (≥10 h)	Moderate	[67]
Cu@Ti_3_C_2_T_x_	High ∼735	High 70%	−0.6	6 h	Moderate	[27]
Ru‐Based Catalysts	High (up to 400)	Low (10–20)		Moderate‐but get poisoned	High cost,	
Cu@V_2_C	High ∼525	High 83%	−0.7	Good (≥10 h)	Moderate	[27]
CuPc@MXene	∼544	High ∼94	−0.4	Good, 56 h	Cheap	[90]
Conventional Cu Catalysts (Cu foil, Cu_2_O)	Average (50–150)	Low (20‐35)		Low‐Moderate (oxidation, agglomeration)	Cheap, readily available	
Ru‐Cu_2_O@Ti_3_C_2_T_x_	∼2180	Moderate 49	−0.7	Good, 24 h	High cost,	[31]
Cu_x_O**/**Ti_3_C_2_T_x_	—	Moderate 48	−0.7			[39]
Cu/Ru@ Ti_3_C_2_T_x_	∼540	High ∼86%	−0.4	Good, 24 h	High cost,	[23]
Ti_3_C_2_T_x_‐Cu sheets	—	High ∼95%	−0.6	Good, 50 h	Moderate	[91]

The electrocatalytic activity of Cu‐based MXenes is highly sensitive to external reaction conditions. The effectiveness and stability of the eNO_x_RR process are ultimately determined by these factors, which also control adsorption/desorption kinetics, charge‐transfer efficiency, and product selectivity. The electrocatalytic performance of Cu‐based MXenes during the eNO_x_RR is significantly influenced by temperature. Improved ionic mobility and rapid interfacial charge transfer accelerate reaction kinetics usually between 25°C and 35°C. Higher NH_3_ production rates and improved FE are the outcomes of this enhancement; the Cu active sites on the MXene surface are better able to absorb and transform NO_x_ molecules into desired products [39, 92]. But it might be harmful to operate at high‐temperatures. Overheating can cause the MXene support to become structurally unstable, sinter or aggregate Cu nanoparticles, and oxidize or delaminate the 2D MXene layers. Furthermore, high‐temperatures can inhibit NO_x_RR and decrease NH_3_ selectivity by accelerating the competing hydrogen evolution process (HER). However, working at lower temperatures (below 25°C) tends to slow down intermediate diffusion and reaction kinetics, thus lowering catalytic activity. For Cu‐based MXenes to remain active and keep their structural integrity during eNO_x_RR, an ideal mild temperature must be maintained [93, 94]. To maintain satisfactory kinetics and retain catalyst integrity, optimal operation is typically maintained at ambient temperatures (∼25°C–35°C). The activity, selectivity, and stability of Cu‐based MXenes throughout the electrochemical reduction of NO_x_ are notably influenced by the electrolyte's pH [17].

## 4 In Situ/Operando Characterization

The mechanism of eNO_x_RR is relatively complex and involves the transport of multiple H^+^/e^−^ via several reaction routes, in contrast to other electrochemical processes like HER, water oxidation, and OER [95]. Cu‐based electrocatalysts may undergo structural redevelopment during electrocatalysis, so it is indispensable to track their surface adsorption states and structural changes in real time to well understand the reaction mechanism, even with significant improvements in the development of effective electrocatalysts [96]. The overall understanding of electrochemical processes is greatly advanced by in situ characterization techniques, especially when it comes to the fabrication of an electrocatalyst for eNO_x_RR that takes into account the variety of intermediate products and reaction routes. In genuine or near‐real reaction environments, these methods enable real‐time assessment of atomic or molecular‐level structural modifications, chemical reactions, and dynamic processes. Consequently, the efficient use of in situ characterization agrees with catalyst refinement for NO_x_RR and provides a window into the internal workings of electrochemical systems.

### 4.1 Electrochemical In Situ Raman Spectroscopy

A detection technique based on light scattering on material surfaces is called in situ Raman spectroscopy [97, 98]. The Raman shift is the frequency difference between the scattered and incident rays. When an incident ray strikes a surface, some photons not only change direction but also change frequency, leading to inelastic scattering at a different frequency [99]. Raman spectroscopy can qualitatively analyze molecular composition because changes in molecular vibrational energy levels determine the Raman shift, and because various chemical bonds and radicals have distinct vibrational energy spectra [100]. It is possible to obtain in situ Raman spectroscopy of dynamic changes in catalyst structure by electrochemically monitoring the reaction in real‐time. A laser transmitter, a focusing microscope, and a signal receiver make up the Raman spectrometer device. In contrast, an electrochemical cell integrated into the Raman system forms the in situ Raman spectrometer. The electrochemical cell setup uses a standard three‐electrode configuration, with a laser microscope used to precisely focus the beam vertically onto the working electrode's surface, which is coated with the catalyst. To reduce signal loss in the electrolyte, the distance between the two electrodes is kept as small as possible. The catalyst is shielded from scattered rays by a quartz window that sits between it and the microscope.

**FIGURE 11:**
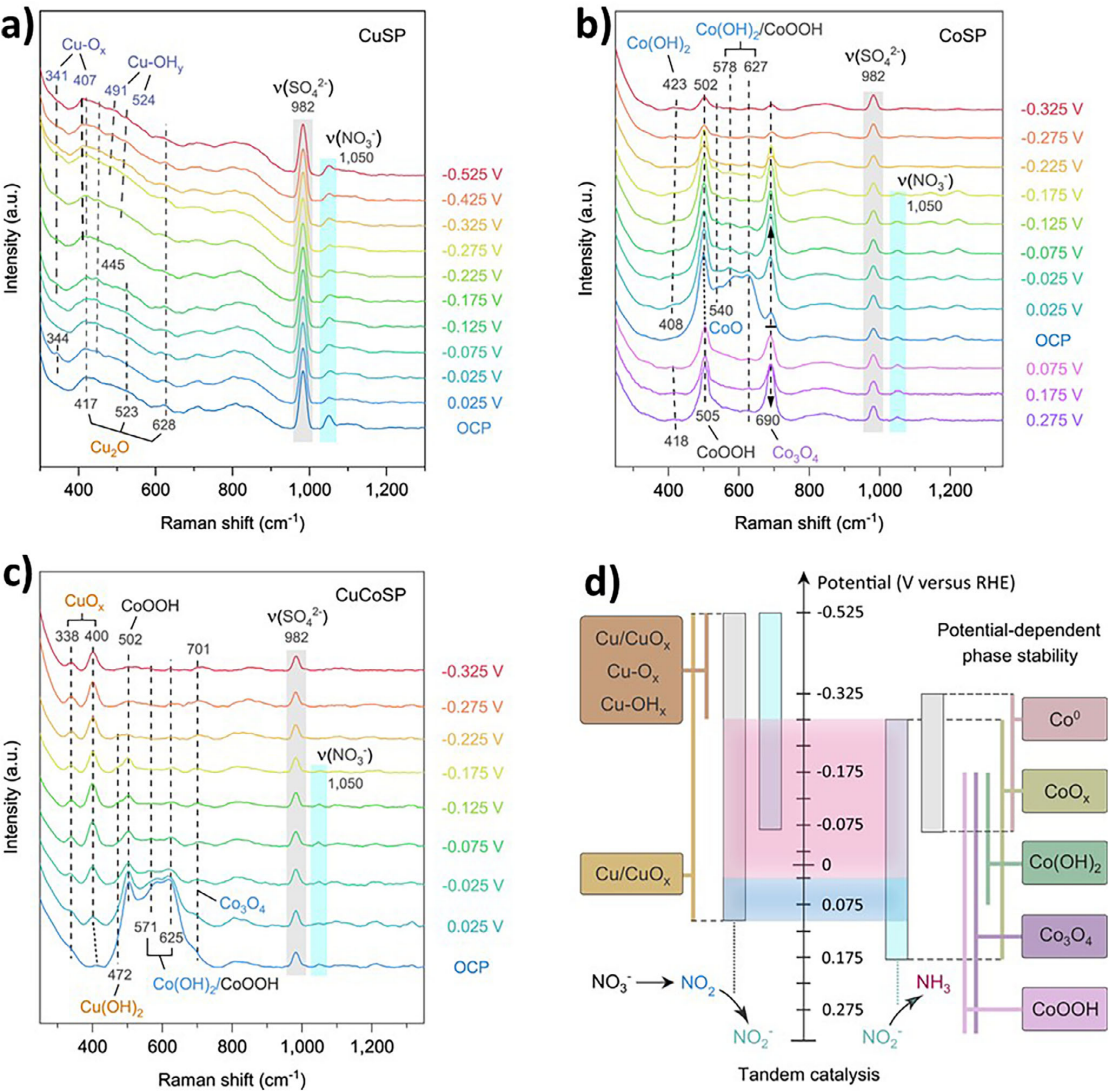
CuSP (a), CoSP (b), and CuCoSP (c) in situ Raman spectra at different applied potentials in electrolytes containing 0.01 m NO_3_^−^, 0.04 m K_2_SO_4_, and 0.01 m KOH. Catalysis of NO_3_RR by CuCoSP tandem at low overpotentials: a suggested reaction mechanism. The possible range for the reduction of NO_3_^−^ to NO_2_ species at the Cu/CuO_x_ phases, which would etch catalysts, is shown by the light blue region in the middle of (d), whereas the potential range for the effective tandem reduction of NO_3_^−^ to NH_3_ is shown by the light pink region, reprinted with permission from Nature Communications [101].

Cyclic Voltammetry (CV) or constant current reaction is performed in the electrochemical cell, and the signal is collected by turning on the Raman spectrometer. During electrocatalysis, Raman spectroscopy uncovers critical information about the catalyst's structure. To examine surface structure reconstruction in Cu─Co binary sulphides (CuCoS), for instance, He et al. used in situ Raman spectroscopy [101]. In situ Raman spectroscopy, kinetic analysis, and electrochemical measurements reveal that the internal Cu/CuOx regions primarily facilitate the initial conversion. This intermediate is then rapidly reduced to NH_3_ on the outer Co/CoO shell, highlighting the synergistic role of the core–shell structure in enhancing catalytic efficiency. Raman spectra of CuSP at decreasing NO_3_RR‐related potentials are shown in Figure 11a. The first wide bands linked to Cu_2_O phases are located at 417, 523, and 628 cm^−1^ and endure as low as −0.525 V [102]. The NO_3_RR has almost no effect on how quickly these Raman peaks change (Figure 11c). The limited influence of NO_3_RR on the phase transformation rate is likely due to the rapid nitrate‐reducing activity of CuCoSP, which facilitates the formation of a nitrate‐rich layer. Unlike CoSP (Figure 11b), applying potentials of 0.025 and −0.025 V in a 0.01 m NO_3_^−^ solution does not enhance the Raman signals associated with Co^3^^+^ phases in CuCoSP. This suggests that on the Cu‐containing regions of CuCoSP, nitrate is efficiently reduced to NO_2_^−^ rather than being oxidized to NO_2_^−^. Consequently, the Cu/CuO_x_ components help stabilize the active Co^2+^ phases, forming a synergistic tandem system that enables stepwise NO_3_^−^ to NH_3_ conversion at relatively low overpotentials (Figure 11d).

Additionally, shell‐isolated nanoparticle‐enhanced Raman spectroscopy broadens the range of surface adsorption and reaction phenomena that can be probed spectroscopically. The resulting Raman signals offer valuable insights into electrocatalyst surface chemistry in the low‐wavenumber region below 750 cm^−1^, as well as detailed information on adsorbed reaction intermediates within the 750–1700 cm^−1^ range. The presence of oxynitride intermediates on the electrode surface leads to the detection of Cu‐O_x_ and Co‐O_x_ structures. Additionally, various species and intermediates such as *NO_3_, *NO_3_, *NO, *NH_2_, *NOH, *NH_3_, NO_3_^−^, and NH_3_ were identified. Among the available techniques, in situ infrared (IR) spectroscopy is the most widely used for probing the chemical bonds of surface‐adsorbed intermediates [103, 104].

Recently, Zhang et al. created sulfur‐doped Cu_2_O (S‐Cu_2_O) nanoneedle arrays via in situ electrochemical processing [105]. They used Raman spectra, X‐ray photoelectron spectroscopy (XPS), and X‐ray diffraction (XRD) to examine the structural change. CuS has a prominent peak in the Raman spectra at 468 cm^−1^, which is associated with S─S bonds in the CuS unit cell [106]. In contrast, following CV scans, the S─S bonds were eliminated. The Cu─O bonds inside the Cu_2_O lattice are responsible for the emergence of peaks at 150, 220, and 635 cm^−1^ [10]. Daiyan et al. developed a CuO electrocatalyst for ENO_3_RA by defect engineering and examined the impact of oxygen vacancy defects in CuO on the adsorption energy in situ Raman and DFT [107]. Using in situ Raman spectroscopy, they were able to demonstrate the genuine active site of CuO_x_ with a distinct CuO‐related peak at wavenumber 300 cm^−1^ in the absence of electrolyte and open‐circuit potential (OCP). However, as the cathode potential shifted to the negative side, the peak gradually vanished, and a new Raman peak corresponding to the Cu_2_O species gradually emerged at 420 cm^−1^. At 300 cm^−1^, however, a CuO signal reappeared after the process ceased. The combination of CuO and Cu_2_O constituted the catalyst's true active site, as demonstrated by in situ Raman spectroscopy, which also revealed that CuO was reduced to Cu_2_O during the NO_3_^−^ reduction process and oxidized to CuO following the reaction. This suggests that the transition was reversible.

Since water has weak Raman scattering, Raman spectroscopy can also be used to characterize aqueous samples. In situ Raman spectroscopy has become increasingly popular in the study of material structure [108]. This spectroscopy does have some limitations, though, such as the inability to characterize many electrocatalysts due to their lack of characteristic Raman peaks and the fact that many reaction intermediates are instantaneous, meaning they are too short to acquire the signal when compared to conventional Raman spectroscopy. As a result, other characterizations must be assisted for analysis.

### 4.2 Electrochemical Operando Vibrational Spectroscopy

**FIGURE 12:**
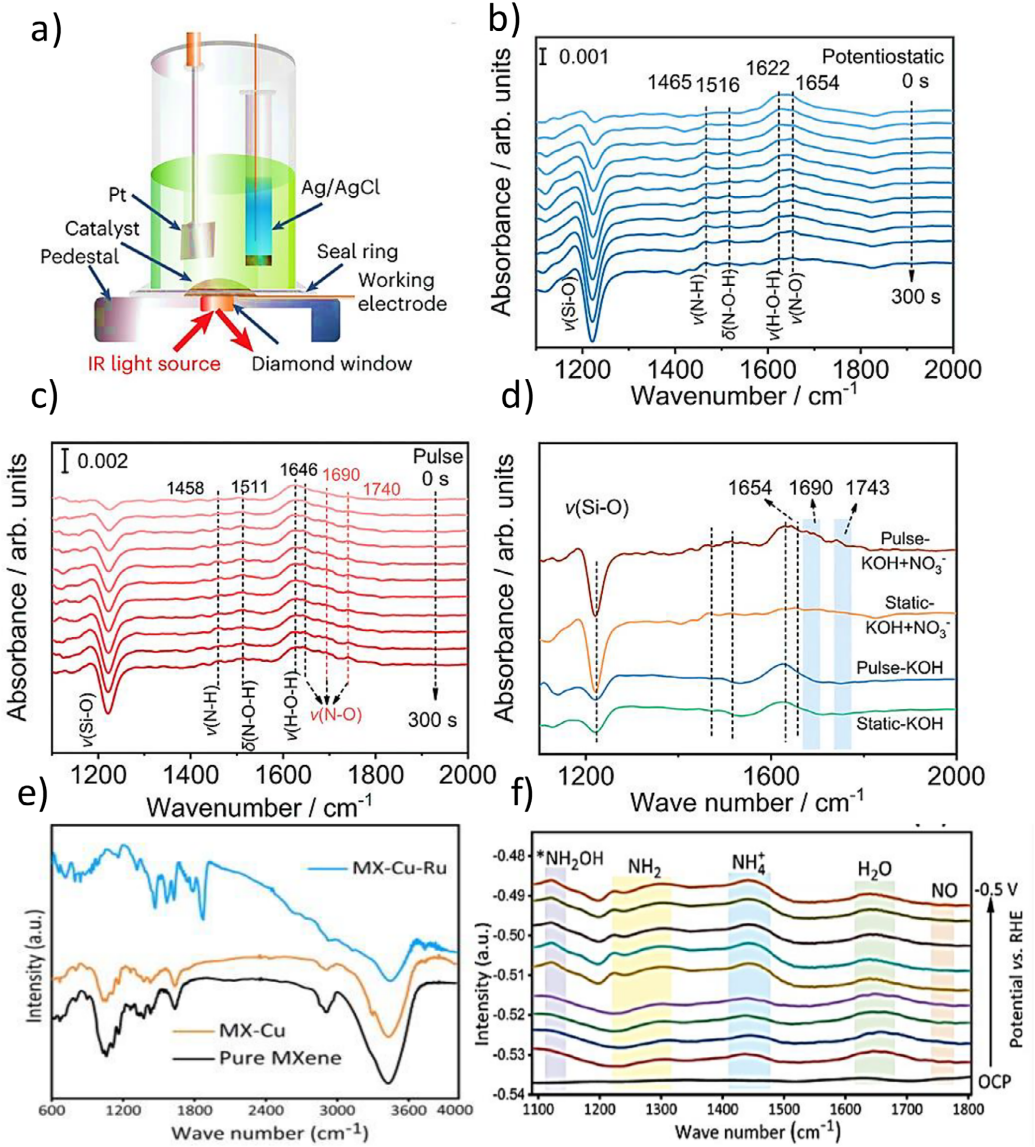
(a) In situ FTIR setup schematic design. Reproduced with permission, reprinted with permission from Nature Sustainability, Springer Nature, copyright 2023 [115]. Time‐dependent ATR‐FTIR spectra of NRA in situ under two different potential conditions: (b) constant (E = −0.1 V) and (c) pulsed (Ec = −0.1 V, Ea = +0.6 V, tc = 4 s, ta = 0.5 s). (d) The comparison of in situ ATR‐FTIR spectra at t = 300 s under various settings [113]. (e) FTIR results of pure MXene, MX‐Cu, and MX‐Cu‐Ru; (f) ATR‐FTIR spectra with prominent peaks of NH_4_^+^, NH_2_, and H_2_O, reprinted with permission from Nano Mat. Sci. (Elsevier) [23].

An Attenuated Total Reflection Fourier Transform Infrared Spectroscopy (ATR‐FTIR) crystal and infrared spectroscopy form the foundation of the ATR‐FTIR sampling method [109]. The main characteristics to choose a particular ATR crystal material are the crystal's wavelength cutoff, refractive index, and chemical stability in a range of pH values. When an infrared beam propagates through an optically dense crystal with a refractive index higher than that of the surrounding medium, total internal reflection at the crystal‐medium interface generates an evanescent field. This evanescent wave extends beyond the crystal surface into the adjacent medium, enabling interaction with species at the interface and in the near‐surface region of the surrounding phase [110]. Nevertheless, it should be mentioned that light can penetrate deeper than both the penetration depth and the dp value. As a result, data obtained from the sample may originate from a depth that is two to three times dp. In situ FTIR in electrochemical measurement is made up of an electrolytic cell and a reflection device; the standard ATR‐FTIR device is depicted in Figure 12a [111]. Improved infrared absorption spectroscopy on the surface, or ATR‐FTIR, can guarantee good mass transfer at the catalyst interface because the working electrode is in direct contact with the light‐conducting prism, which is filled with a conductive film material containing catalysts [112]. Through a prism window, the catalysts are illuminated by infrared radiation. Internal reflection then takes place where two media with different refractive indices meet, thereby enabling the transmitted radiation to be detected and converted into an infrared spectrum [110].

The ATR‐FTIR is frequently employed for the identification of adsorption intermediates between the electrolyte and the catalyst owing to its exceptional sensitivity and its ability to prevent interference from the electrolyte's aqueous solution. The in situ ATR‐FTIR system configuration is depicted in Figure 12b [108]. In particular, an electrochemical cell using a single‐bounce silicon crystal modified with an Au film is used to measure under internal reflection conditions with a spectroscopic system. The fabrication of the Au‐coated, single‐bounce silicon crystal was carried out before performing electrocatalytic experiments (Figure 12c) [113]; (1) Submerged the monocrystal silicon in aqua regia for 20 min and then polished it with Al powder for 10 min after washing it three times with ultrapure water and acetone; (2) Submerged the aforementioned monocrystal silicon in a mixture of H_2_SO_4_ and H_2_O_2_ (Vconcentrated H_2_SO_4_: VH_2_O_2_ = 1:1) for 20 min; (3) Submerged the monocrystal silicon three times with water and then three times with ultrapure water; (4) Submerged the monocrystal silicon in a mixture of 15 mL of solution A, which contained NaOH, NaAuC_l4_·2H_2_O, NH_4_Cl, Na_2_SO_3_ Na_2_S_2_O_3_·5H_2_O, H_2_O, and 3.4 mL of 2% NH_4_F aqueous solution; The absorbed intermediates are then monitored using electrochemical in situ ATR‐FTIR. Absorbance values are used to depict peak intensity; positive bands indicate the formation of reactive intermediates, while negative bands indicate their consumption. In situ ATR‐FTIR spectroscopy may successfully detect the groups generated on the surface of the catalyst by considering the absorption peak location and intensity, which correspond to the molecular structure and the number of chemical groups, respectively [114]. This provides more information on the catalytic process and helps speculate about the reaction intermediates.

The routine FTIR analyze the vibrational modes of chemical bonds in a sample. Because it records the IR absorption spectrum, ATR‐FTIR is often used because it eliminates the need for pellet formation (e.g., KBr pellets) and reduces sample handling. Using in situ ATR‐FTIR, the electroreduction process over n‐HEA was investigated [116]. Hu et al. used in situ ATR‐FTIR to track the reactant and intermediate adsorption configuration at 0.01 and 0.1 m nitrate [117]. Ru/Cu_2_O exhibits *NO, and *NH_2_OH species at high concentrations of 0.1 m. Ru/Cu_2_O FTIR spectra showed peaks linked to *NO_2_, *NHO, and *NH_3_ when the concentration was lowered to 0.01 m. This was caused by Ru nanoparticles' ability to favor hydrogen transfer by nitrate ions by matching the pace of HER and NO_3_^−^RR kinetics.

To better understand the catalytic mechanism, optimize the reaction conditions, and develop Ru‐based catalysts for increased NH_3_ synthesis, in situ ATR‐FTIR provides real‐time, molecular‐level information on species during NO_3_^−^RR. Another recent research uses FTIR to demonstrate the incorporation of Ru into the Cu‐MXene [23]. FTIR analysis (400 cm^−1^ to 4 000 cm^−1^) verifies the presence of surface functional groups and confirms the successful synthesis of MXene, Cu‐MXene, Ru‐MXene, and Ru/Cu‐MXene catalysts (Figure 12d). Distinct absorption bands observed at approximately 640 cm^−1^ and 700 cm^−1^, assigned to O─Ti─O/Ti─O and Ti─O─Ti, respectively. A pronounced band appearing in the 1650–1690 cm^−1^ region corresponds to C═O stretching, indicating the presence of surface‐terminating groups such as = O, ─F, and ─OH that are beneficial for catalytic activity. These features further confirm that Al layers are effectively removed during the etching process and replaced by oxygen‐ and fluorine‐containing functional groups. Additionally, absorption bands in the range of 1100–1500 cm^−1^ are associated with the Cu‐MXene, Ru‐MXene, and Ru/Cu‐MXene composites. The absence of distinct characteristic peaks for Ru or Cu species suggests low loading and high dispersion of these species on the MXene surface [39].

In situ external reflection FTIR spectra were recorded on the Ru/Cu‐MXene electrode from OCP to −0.5 V vs. RHE as described in Figure 12e,f. It reveals the consumption of NO species (1740–1780 cm^−1^) and the simultaneous formation of hydrogenated intermediates, including NH_4_^+^ (∼1450 cm^−^^1^), *NH_2_ (∼1310 cm^−1^), and NH_2_OH (∼1170 cm^−1^). The increasing H─O─H bending signal (1650–1700 cm^−1^) indicates water electrolysis, which supplies reactive H species to drive the stepwise reduction of NO/NO_x_ toward NH_3_. Apart from identifying reaction intermediates, in situ ATR‐FTIR shows significant promise for examining dynamic changes in the adsorbate under reaction conditions, which in turn alter its catalytic behavior [118]. However, in situ ATR‐FTIR spectroscopy results in aqueous‐phase investigations are frequently very weak due to the sensitivity of IR light to H_2_O molecules, which leads to modest signal‐to‐noise ratios.

### 4.3 Electrochemical In Situ Electron Paramagnetic Resonance (EPR) Measurement

**FIGURE 13:**
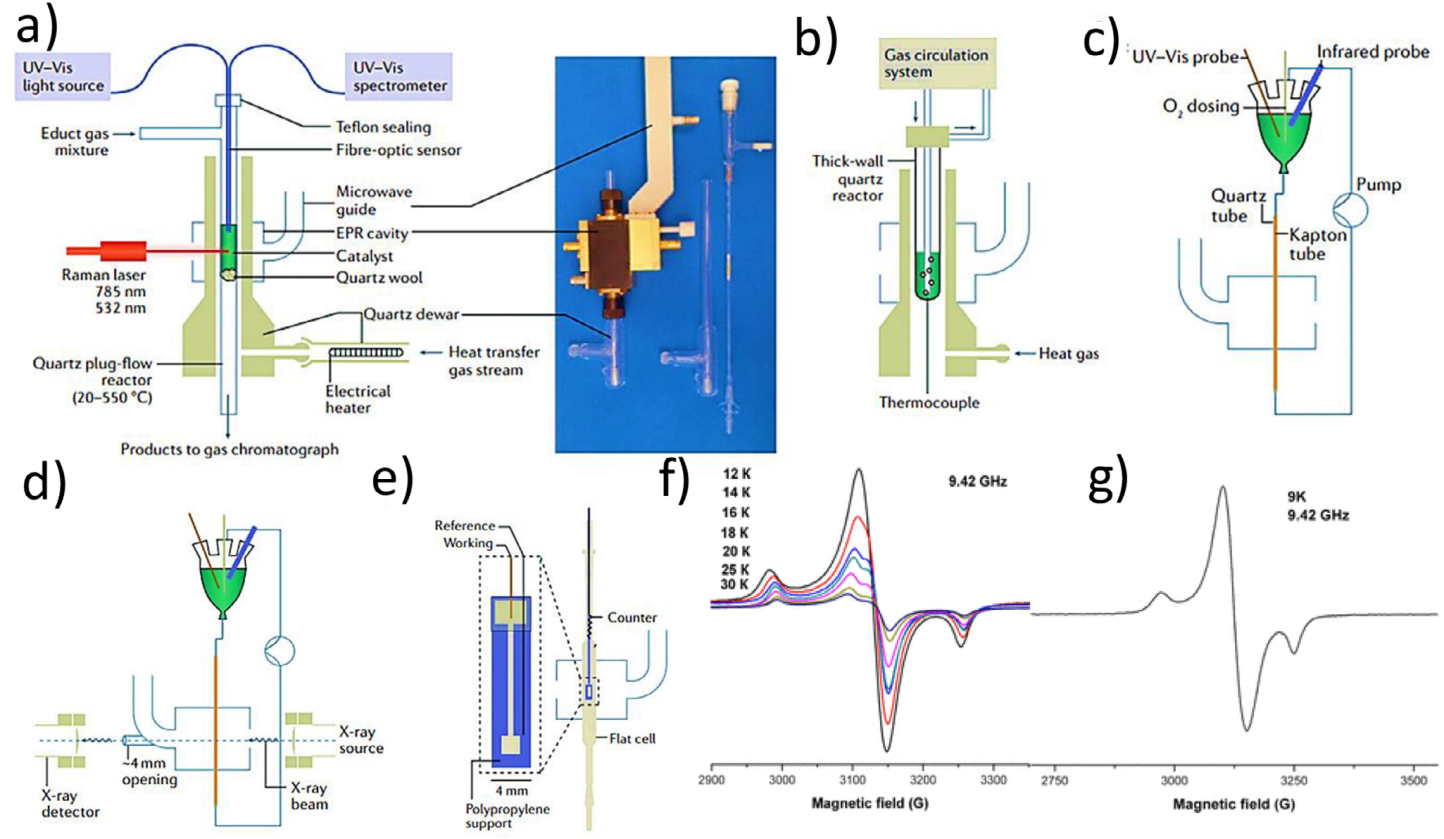
In situ EPR measurement experimental setups: (a) A quartz flow reactor intended for the analysis of heterogeneous catalysts in solid‐gas phase reactions at pressures of up to 20 bar and temperatures of up to 500°C. (b) A quartz batch reactor with a thick wall that can handle gas‐liquid phase reactions up to 20 bar and 100°C. For coupling with X‐ray absorption spectroscopy, a homogeneous reaction tank with a quartz tube and pump is used to cycle the solution through the EPR resonator without (c) and (d). (e) A flat cell including a standard three‐electrode arrangement for EPR Spectro electro chemistry, Nature Reviews Methods Primers [119]. Conduct of the ESR spectra of the copper (II) complex with functionalized hyperbranched polymer BH_3_0 (f) at 9 K and (g) between 30 and 12 K, reprinted with permission from ACS Omega [122].

Throughout catalytic cycles, numerous catalysts pass through paramagnetic intermediates that contain unpaired electrons, providing valuable insight into their electronic configurations and local structures. Several responses also follow radical processes. By directly probing these unpaired electrons, electron paramagnetic resonance (EPR) spectroscopy can determine the oxidation states and coordination geometries of transition‐metal ions and characterize molecular radicals [119]. In essence, in situ electron spin resonance (ESR), another name for EPR, is a spectroscopic method based on the resonant absorption of microwave radiation energy linked to unpaired electron spin level transitions [120]. These systems contain species with unpaired electrons that may be utilized to sensitively study their electrical and geometric structure. These unpaired electrons can be directly studied by EPR spectroscopy, which enables the creation of active sites during redox processes, the identification of transition metal ion oxidation states, and the characterization of molecular radicals [121]. Figure 13a–g provide schematic illustrations of common experimental configurations used to measure EPR in situ. This method enables the identification of active redox pathways.

The primary use of in situ ESR is the detection of free radicals in solution; however, it has also been used in certain studies to investigate free radicals on the electrode surface and electron spins within the electrode material. In situ ESR is frequently employed to identify the presence or alteration of hydrogen radicals (*H) in reduction processes. The quantity of spin is estimated using peak area, and ESR is quantified at the spin level using relative or semi‐quantitative methods rather than absolute ones [120]. Peak position (g value), number of subpeaks, and hyperfine splitting constant (horizontal distance between specific subpeaks) are used to ascribe a peak, which is frequently regarded as a pharmacological fingerprint [123].

The lifetime of radical‐radical coupling is typically very short (5–30 nanoseconds), while magnetic field scanning in ESR measurements takes several seconds. It should be noted that only stable free radicals can be identified by ESR, even though in situ ESR is a crucial characterization tool for identifying free radicals during a reaction [123]. Furthermore, the NO_x_ RR mechanism is mediated in large part by hydrogen radicals. Therefore, studying hydrogen radicals is necessary to comprehend the genesis of NO_x_ reduction. Tensile lattice strains enable hydrogen radicals to lower the kinetic barriers to hydrogenating intermediates into NH_3_, which accelerates the conversion of NO_x_ to NH_3_. Because of its excellent sensitivity to concentrations at ≈10–12 mol, in situ EPR spectroscopy has been widely employed to identify the radicals produced on catalyst surfaces [124]. Consequently, by using in situ EPR spectroscopy, we may get conclusive knowledge on the hydrogenation process that takes place during NO_x_RR, improving our overall comprehension of the underlying reaction route and processes. The typical characteristic peaks of *H are displayed as a series of peaks with a peak height in the ESR result, indicating that there are *H in the system at this time. In situ ESR can also detect *H during the reaction, and the use of DMPO as a trap agent can capture the *H produced in the reaction. The reaction may be classified as a direct or indirect reduction mechanism based on the combination of the in situ ESR data, changes in the products following the reduction, and the employment of *H as a reducing agent. Considering how intense the peaks are, the in situ ESR measurements may also be utilized to compare the catalyst's adsorption ability to *H. Researchers can better grasp the reduction mechanism of NO_x_ in reactants by using a mix of in situ approaches to visualize the conversion of reactants in a more comprehensible manner.

### 4.4 In Situ X‐Ray Absorption Spectroscopy (XAS)

X‐ray absorption spectroscopy (XAS) is a technique for determining the element‐specific local geometric and electronic structure around the absorbing element. For simplicity, XAS is divided into two main regions: the extended X‐ray absorption fine structure (EXAFS) and the X‐ray absorption near‐edge structure (XANES), mostly because of the interpretation of the data. We limit our discussion to the more common use of hard X‐rays (over 3000 eV), employed in in situ/operando electrocatalysis investigations. Transmission and fluorescence are the two main methods by which XAS collects more realistic data. The most common method is transmission, which uses Beer's law to measure the X‐ray energy before and after it passes through the material.

It is widely reported that this mode poses difficulties for electrochemical investigations, as the electrolyte frequently absorbs a large portion of the X‐ray intensity, and the cell design must allow X‐rays to pass through the cell and the substance without altering the X‐rays that are released. The fluorescence signal emitted by the absorber element is collected in the fluorescence yield mode. This is by far the most common way to get electrocatalyst XAS data [125]. This method offers information on the catalysts' electronic configurations, oxidation states, and active sites throughout the reaction, which are essential for connecting structure to function. In light of this, in situ XAS has advanced quickly in recent years.

Lately, Wang et al., believed that because of their advantages and suitable intrinsic activity, Nitrate might be electrocatalytically reduced to NO_3_RR using single‐atom catalysts (SACs) based on copper. In this work, low‐coordinated Cu‐N_3_ single‐atom catalysts (SACs) are synthesized and anchored onto high‐curvature, hierarchically porous nitrogen‐doped carbon nanotubes (NCNTs) using a sequential approach involving polymerization, surface modification, electrostatic adsorption, and carbonization [21]. Unlike conventional SACs and Cu‐based catalysts, the resulting Cu‐N_3_ SACs/NCNTs exhibit outstanding activity for nitrate reduction, achieving a peak Faradaic efficiency of 89.64% and an NH_3_ production rate of 30.09 mg per mg of catalyst per hour (equivalent to 70.8 mol g Cu^−^^1^ h^−^^1^). In situ X‐ray absorption spectroscopy (XAS) was employed to monitor the structural evolution of Cu‐N_3_ SACs/NCNTs during the NO_3_^−^ reduction process.

**FIGURE 14:**
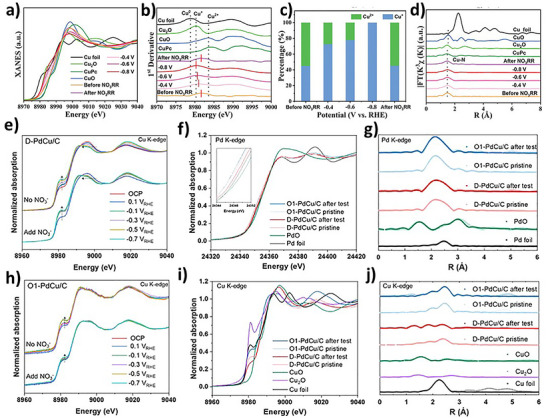
XAS measurements in situ. (a) Cu K‐edge XANES spectra, (b) First‐derivative XANES curves, (c) Cu K‐edge XANES spectra LCF result, and (d) Cu‐N_3_ SACs/NCNT FT‐EXAFS spectra, reprinted with permission from Adv. Fun. Mat. (Wiley) [21]. XAS characterization of D‐PdCu/C and O1‐PdCu/C electrocatalysts. (e, h) In situ Cu K‐edge XAS spectra collected during NO_3_^−^ reduction with and without nitrate in 0.05 m Na_2_SO_4_ electrolyte. (f, i) Normalized XANES spectra at Pd and Cu K‐edges recorded before and during extended electrolysis; (g, j) Corresponding Fourier‐transformed EXAFS spectra at Pd and Cu K‐edges derived from (f) and (i), respectively, revealing changes in local atomic structure, reprinted with permission from ACS Energy Letters [126].

As shown in Figure 14a, in contrast to the fresh catalyst, the XANES spectra clearly demonstrate a drop in edge energy as the applied voltage increases. The XANES spectra were fitted using linear combination fitting (LCF) and the first derivative to more clearly show the spectrum development and determine precise percentages of Cu species with various valence states (Figure 14b,c). 44.9% of the new catalyst is Cu^+^ and 55.1% is Cu^2+^_,_ suggesting a condition of dual valence. The positions of the peaks gradually shift toward lower energy as the applied potential rises, and the ratio of Cu^+^/Cu^2+^ declines, signifying a decline in Cu species. The catalyst is fully composed of Cu^+^, and the Cu^+^ feature dominates the first derivative of the XANES spectrum at ‐0.8 V in relation to RHE (the potential for achieving the greatest FENH_3_). Following NO_3_RR, the Cu species compositions are almost identical, and the related first derivative and XANES spectra return to their original shape. Furthermore, in accordance with XANES spectra, the related FT‐EXAFS spectra further validate the preservation of Cu‐N coordination at various potentials and the lack of Cu─Cu bond (Figure 14d), The preservation of Cu‐N is further confirmed by the related FT‐EXAFS spectra [21]. According to the in situ XAS data, the Cu‐N_3_ SACs/NCNT shows a dynamic structural development during the reaction, and as the applied potential increases, so does the ratio of Cu^+^/Cu^2+^. Cu‐N_3_ SACs/NCNT's almost identical XANES and FT‐EXAFS spectra before and after NO_3_RR show that the catalyst is stable.

In a similar vein, Lim et al. reveal that an electrocatalyst based on a highly ordered PdCu alloy offers almost total (94%) nitrate elimination without catalyst loss, stability (480 h), and selectivity (91% N_2_) [126]. Enhanced structural integration between Pd and Cu contributes to improved catalyst durability during the nitrate reduction reaction (NO_3_RR), as confirmed by both in situ and ex situ X‐ray absorption spectroscopy (XAS). This method was employed to track variations in oxidation states and the local atomic arrangement around Pd and Cu sites. When D‐PdCu/C was thermally treated at 500°C to produce O1‐PdCu/C, the Cu‐Cu coordination number declined from 6 to 4.8, while the Pd‐Cu coordination number increased from 2.4 to 3.4. Moreover, XANES analysis under negative potentials ranging from 0.1 to −0.7 V vs. RHE showed that CuO_x_ species gradually reduced to metallic copper, regardless of the catalyst composition or the presence of nitrate ions (Figure 14e–j). This study indicated that they are more dominant than the oxidation of the Cu sites. Pd K‐edge spectra for ex situ measurements showed no differences between the D‐PdCu/C and O1‐PdCu/C catalysts before and during the long‐term NO_3_RR operations (Figure 14f,g). However, the ex situ characteristics of the Cu K‐edge during long‐term electrolysis are impacted due to the elevated Cu oxidation states in comparison to the pristine catalyst (Figure 14i,j). D‐PdCu/C revealed a substantial shift. The O1‐PdCu/C spectra, however, remained unchanged, suggesting that the oxidation states were consistent both before and after prolonged electrolysis [126]. The strong stability of intermetallic Pd and Cu nanoparticles over extended NO_3_RR operations was shown by various O1‐PdCu/C stable states.

Recent work has shown that catalysts must be evaluated under conditions that closely simulate their actual operating environment, especially in the presence of water, which strongly influences their behavior [127]. In this context, Günter and co‐workers investigated the NH_3_‐SCR of NO_x_ using advanced X‐ray emission and absorption techniques [128]. XAS is widely used to probe the local electronic and structural surroundings of metal sites, including Cu anchored on MXene supports for electrochemical NO_x_ reduction. However, conventional XAS approaches, whether in transmission or fluorescence mode, struggle to differentiate between atoms with similar scattering properties, such as nitrogen and oxygen, which limits the ability to unambiguously identify surface ligands formed during reaction. Moreover, the XANES portion of the spectrum suffers from significant core–hole lifetime broadening, which masks fine spectral details associated with transitions into unoccupied electronic states [129].

One strategy to overcome this limitation is to record absorption features through fluorescence lines with much longer lifetimes, giving rise to high‐energy‐resolution fluorescence‐detected (HERFD) XANES. Günter et al. demonstrated that combining HERFD‐XANES with valence‐to‐core X‐ray emission spectroscopy (V_2_C‐XES) greatly enhances sensitivity to changes in oxidation state, coordination structure, and ligand identity compared with standard XAS. Their work also showed that these techniques can visualize gradients in metal oxidation and ligand coverage along a catalyst bed during NO_x_ conversion. This level of detail is essential for distinguishing Cu^+^ from Cu^2+^, identifying linear vs. higher‐coordination geometries, and determining whether species such as NH_3_ or NO are bound to the active centers information that directly informs mechanistic understanding.

The same reasoning applies to electrochemical NO_x_ reduction on Cu‐MXene catalysts but with two key differences. In an electrochemical system, the primary variable is the applied potential, coupled with local variations in electrolyte composition, rather than position in a reactor bed. Tracking XANES or HERFD‐XANES as a function of potential can therefore capture transitions among Cu^2^^+^, Cu^+^, and Cu^0^, as well as changes in coordination enforced by MXene terminations (─O, ─OH, ─F) or bound intermediates such as *NO or *NH_x_. In parallel, V_2_C‐XES can discriminate between N‐ and O‐based ligands and can reveal whether Cu is coordinating NO, NH_3_, or protonated NH_x_, providing mechanistic insights similar to those gained in zeolite Cu systems.

Applying the Günter et al. methodology to Cu‐MXene systems enables operando, potential‐dependent mapping of (i) Cu oxidation and coordination states, (ii) the identity and bonding mode of adsorbed intermediates, and (iii) the structural stability of isolated Cu atoms or clusters under reducing conditions. Two spectroscopic signatures are particularly important: the pre‐edge and rising‐edge features in HERFD‐XANES, which reflect Cu oxidation and coordination environment, and the ligand‐sensitive V_2_C‐XES fingerprints that differentiate between O‐ and N‐coordinated adsorbates and their protonation states. Together, these measurements clarify whether MXene terminations help stabilize the preferred Cu state, whether strongly bound *NO/*NH_x_ species accumulate on the surface, and whether regeneration steps (such as pulsed electrochemical operation) restore the catalyst to its original condition. The interpretive framework established by Günter et al. provides a strong foundation for extending operando X‐ray spectroscopy into electrochemical NO_x_‐to‐NH_3_ systems.

Instead of having a well‐ordered crystalline structure, the catalyst surface that coexists with reactants, intermediates, and products in the actual electrocatalytic process is usually characterized by disordered characteristics. XANES and EXAFS spectra can provide unique information on the electronic configuration and coordination around the atoms, respectively, to the electrocatalysts lacking long‐range order [130, 131]. The behavior of Ru‐based electrocatalysts under real‐world working conditions and their reaction mechanism can be deeply understood thanks to in situ XAS, which can show the interactions between reaction intermediates and Ru‐based catalysts as well as the dynamic evolution of the reaction process. This information is crucial for improving electrocatalytic NO_x_ RR's FE and selectivity as well as for improving catalyst design. The in situ XAS strategy only offers a mediocre description of internal structures, despite its significant importance. This constraint confines its broader applicability by making it difficult to utilize for component analysis for complex systems. Key elements are labeled, including the precise target of each technique (e.g., intermediates or catalysts), the kind of information gathered (e.g., structural, electrical, or chemical bonding data), and the pros and cons of each method. This comparative review is a useful guide for choosing the right instruments for eNO_x_RR studies and aids in finding relevant methods to evaluate various facets of the electrocatalytic process, such as intermediate production, catalyst surface modifications, or dynamic structure evolution.

**FIGURE 15:**
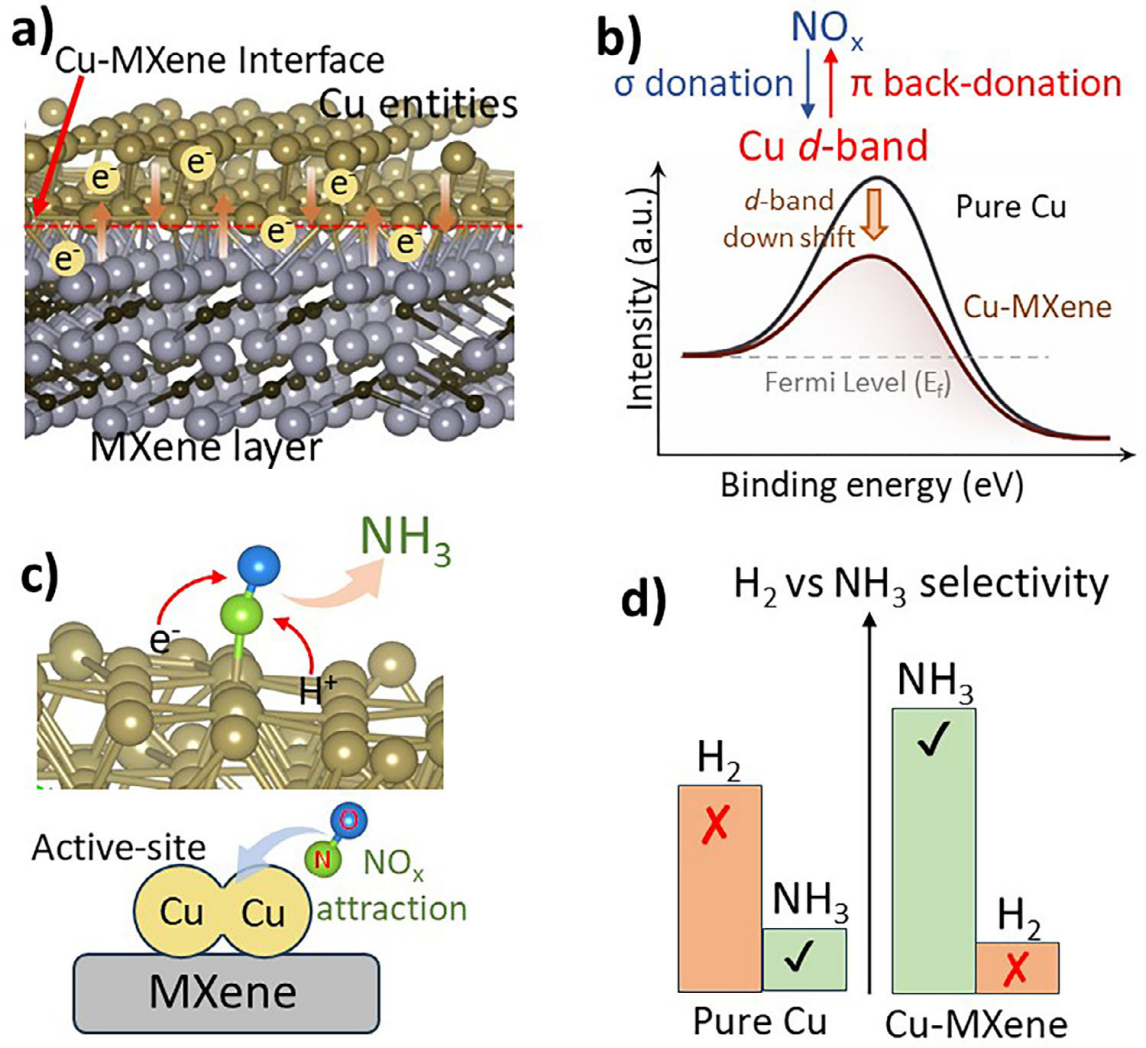
Illustration of Cu‐MXene interfacial effects during NO_x_ electroreduction. Electron transfer from MXene to Cu tunes the d‐band, enhancing NO_x_ adsorption, proton‐coupled electron transfer, and selective NH_3_ formation. The schematic highlights superior NH_3_ selectivity of Cu‐MXene compared with pure Cu, emphasizing the role of interfacial electronic interactions.

Building on the operando XAS insights into Cu oxidation states and coordination dynamics, Figure 15 schematically depicts how the Cu‐MXene interface modulates electronic interactions, NO_x_ adsorption, and H^+^‐coupled e^−^ transfer, collectively governing selective NH_3_ formation. Electron transfer from the conductive MXene support to Cu active sites tunes the Cu *d*‐band, enabling synergistic σ‐donation and π‐back‐donation with adsorbed NO_x_ intermediates. This interfacial electronic modulation strengthens reactant adsorption, stabilizes intermediates, and suppresses competing hydrogen evolution, thereby promoting selective proton‐coupled hydrogenation toward NH_3_. Stepwise e^−^ and H^+^ transfers illustrate the mechanistic progression from NO_x_ adsorption, activation, and hydrogenation to NH_3_ formation. The accompanying selectivity panel highlights the superior NH_3_ production of Cu‐MXene relative to pure Cu. Collectively, this schematic captures how atomic‐scale engineering of Cu‐MXene interfaces orchestrates reaction pathways, providing a conceptual blueprint for designing advanced electrocatalysts for sustainable ammonia synthesis.

## 5 Future Prospects

**FIGURE 16:**
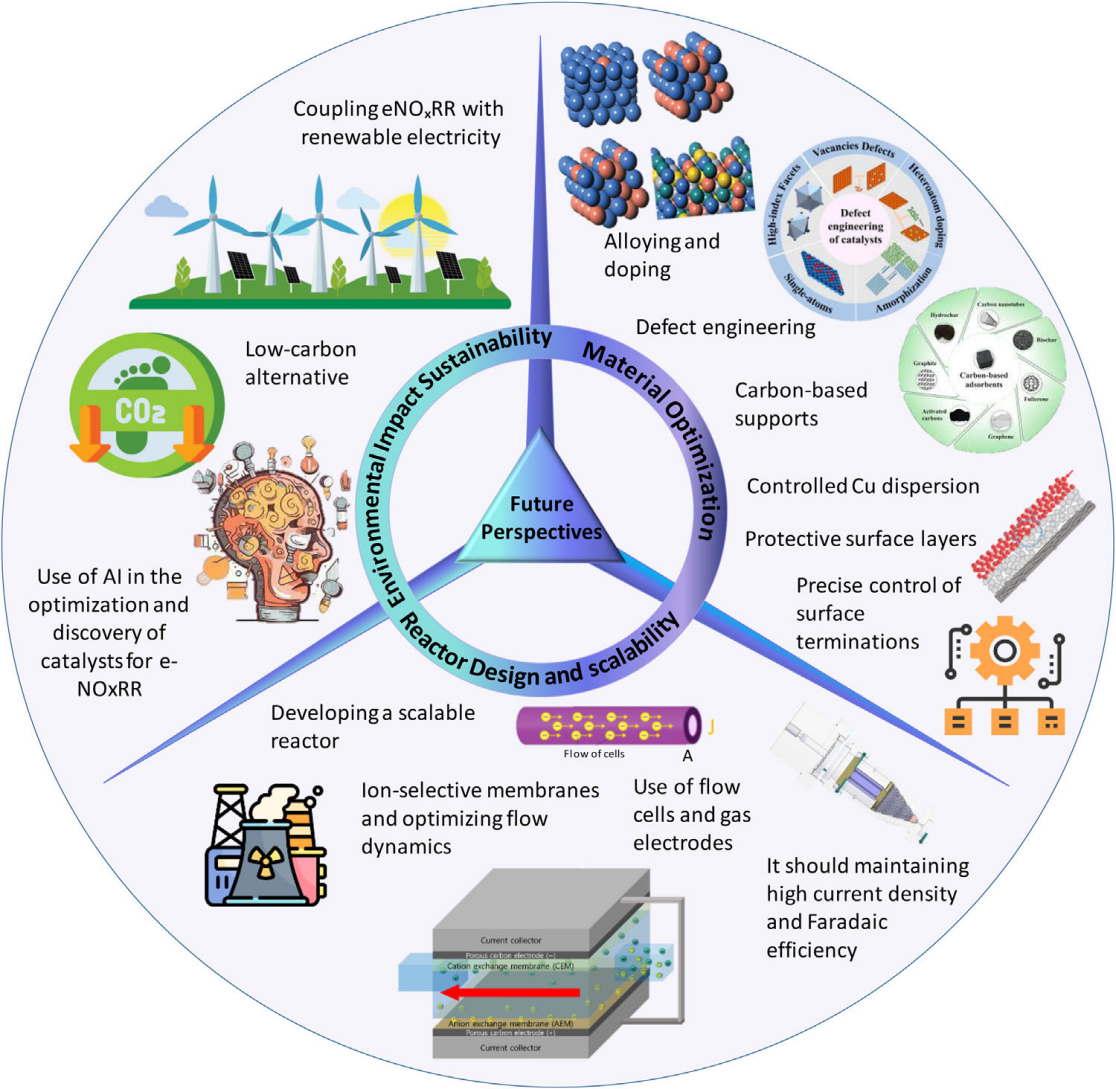
Integrated overview of future perspectives for Cu‐MXene‐based electrocatalysts in NO_x_‐to‐NH_3_ conversion, reprinted with permission from Energies; Membranes (MDPI); Materials Today Sustainability [132, 133, 134].

The electrochemical conversion of NO_x_ to NH_3_ using Cu‐decorated MXene catalysts offers a promising pathway for sustainable NH_3_ harvesting. Although recent developments have significantly strengthened the scientific foundation of this technique, the transition from laboratory‐scale achievement to industrial deployment remains hindered by multiple persistent bottlenecks. These matters span catalyst durability, system's competency, scale‐up limitations, mechanistic doubts, economic viability, and the integration of green energy sources. A summary of future implications is depicted in Figure 16.

### 5.1 Material Optimization

A critical barrier to the practical application of Cu‐MXene systems for eNO_x_RR is the lack of long‐term catalyst durability [135, 136, 137]. Cu‐species are prone to oxidation, surface reconstruction, and erosion under electrochemical conditions, compromising their catalytic performance. Similarly, MXenes, particularly Ti_3_C_2_T_x_, suffer from structural instability due to self‐stacking and oxidation, which limits the accessibility of active sites and lowers electrical conductivity [87]. These degradation pathways not only decrease catalytic efficiency but also hinder scalability [138]. To overcome these constraints, research has focused on integrating stabilizing approaches such as material optimisation, alloying, defect engineering, and the use of carbonaceous materials like reduced graphene oxide (rGO) [139, 140, 141]. For example, Cu/rGO systems have verified high NO_x_ removal rates above 90% even after multiple electrolysis cycles, highlighting the role of support materials for consistent performance [142]. The rational synthesis of heterostructures and the introduction of protective surface layers can also mitigate MXene oxidation. Moreover, the stabilization of catalytically active Cu^+^‐species, critical for intermediate formation in eNO_x_RR, remains a primary hurdle, as Cu^+^ is intrinsically unstable under reducing environments. Studies on heterostructures such as CuAgO_x_ and other single‐atom alloys offer promising pathways for stabilizing these reactive intermediates over extended operational cycles. Furthermore, strategies like alloying [143], defect engineering [144, 145], and exploiting metal‐support interactions (MSI/EMSI) [146] facilitate improvements in catalytic activity and structural maintenance. Optimizing synthesis (e.g., modifying precursor proportions), shielding the surface (e.g., antioxidants or encapsulation), and developing derivation techniques to produce heterostructures with more active sites are all obligatory to address MXene degradation [147, 148].

To achieve high NH_3_ selectivity via suppressing the HER remains one of the most significant hindrances in eNO_x_RR. The efficiency of NO_x_‐to‐NH_3_ conversion is highly dependent on the electronic configuration of active sites and the structural environmental surroundings. Proper surface modifications in MXenes (e.g., ─F, ─OH, ─O) play a major role in adjusting surface reactivity, hydrophilicity, and intermediate adsorption performance. However, striking a balance between improved activity and undesired side reactions, i.e., HER, remains difficult, particularly when high Cu loading leads to excessive e^−^ utilizations. Current research is focusing on designing multifunctional active sites through techniques such as heteroatom doping, the creation of low‐coordination centers, and the introduction of dual‐site catalysts. These designs are inspired by biological enzyme systems that use cooperative active centers to lower the energy barriers and improve product selectivity. Additionally, controlling Cu content and its spatial distribution is essential; insufficient loading results in sluggish NO_x_ conversion, while excessive Cu promotes HER and reduces NH_3_ yield.

### 5.2 Reactor Design and Scalability

Despite promising lab‐scale demonstrations, the practical deployment of eNO_x_RR encounters obstacles in reactor engineering and scalability. Most studies are performed under ideal circumstances using static electrolytic cells, which do not reflect real‐world operating environments [149]. There is a critical need to develop scalable reactor architectures that can efficiently handle gaseous NO_x_ while maintaining high current density and FE. Flow‐cell designs and gas diffusion electrodes (GDE) provide potential for improved mass transport and improved reactant accessibility at the catalyst interface. In addition, integrating ion‐selective membranes and optimizing flow dynamics are essential to reduce mass transfer limitations. System design must also accommodate continuous NH_3_ harvesting and real‐time surveillance to ensure operational stability over extended time spans.

A further constraint lies in the limited understanding of the complicated reaction mechanisms involved in eNO_x_RR. The conversion process involves multi‐step e^−^/H^+^ transfer pathways, making it difficult to determine the nature of active intermediates and rate‐limiting steps. Advanced in situ and operando methods, such as FTIR, Raman spectroscopy, TEM, and X‐ray absorption spectroscopy (XAFS), are needed to trace the catalyst transformations and monitor intermediate species in real‐time [150]. These approaches are essential for mapping structure‐activity relationships and confirming theoretical predictions derived from computational simulations. Combining in situ characterization with DFT calculations can significantly improve mechanistic interpretation and support rational catalyst design.

### 5.3 Environmental Impact Sustainability

Electrocatalytic systems for NO_x_ conversion must also be integrated with renewable energy sources to ensure true sustainability. Conventional NH_3_ synthesis via the Haber‐Bosch process relies heavily on fossil fuels, contributing significantly to global greenhouse gas emissions [151]. By contrast, eNO_x_RR powered by renewable energy (solar, wind, hydro) offers a low‐carbon substitute with modular, decentralized, and feasible potential. This is particularly beneficial in agricultural regions, where on‐site NH_3_ production can support fertilizer demand, reducing transportation costs and discharges. Moreover, using NO_3_^−^‐rich wastewater as a feedstock allows simultaneous pollutant removal and resource recovery, advancing the principles of regenerative economy and environmental remediation.

Artificial intelligence (AI) is emerging as an effective and powerful tool for accelerating the discovery and optimization of eNO_x_RR catalysts [30, 86]. Traditional catalyst development approaches rely on trial‐and‐error investigation, which is time‐consuming and resource‐intensive. Machine learning algorithms can analyze vast datasets from experimental and computational studies to recognize key patterns linking catalyst structure to performance. These models enable rapid screening of materials, estimation of reaction behavior, and optimization of working environments without wide‐ranging laboratory analysis. AI can also provide insights into catalyst degradation mechanisms, thereby improving the stability and reusability of design systems. In catalyst discovery, AI‐driven high‐throughput screening and autonomous learning from DFT databases can expose innovative materials with required properties, supporting the shift from empirical exploration to data‐guided innovation.

## 6 Conclusion and Future Prospects

The electrochemical NO_x_‐to‐NH_3_ process offers a dual solution: mitigating harmful air pollutants and producing NH_3_ sustainably. Compared with direct N_2_ reduction, NO_x_ electroreduction is kinetically more favorable and operates under mild conditions, enabling integration with renewable electricity. Cu‐MXene hybrid catalysts are particularly promising, combining Cu's high NO_x_ reactivity with MXene. Its high conductivity and tunable surface chemistry enable precise control of adsorption, intermediate stabilization, and reaction pathways for selective NH_3_ synthesis.

Catalytic performance is governed by the synergy between Cu active sites and the MXene support. Surface terminations, defects, heteroatom doping, and interfacial electronic effects strongly influence the stabilization of NO_x_‐derived intermediates and the suppression of hydrogen evolution. At the same time, operational parameters, such as electrolyte composition, pH, applied potential, and NOx concentration, have a pronounced impact on selectivity and efficiency. These findings highlight the need for a holistic design approach that integrates engineering, structural tuning, and system‐level optimization. Such an approach is essential to achieve high‐efficiency, selective, and robust NH_3_ production under practical electrochemical conditions.

Despite significant progress, several challenges remain: limited operating windows, modest current densities, and short‐term studies. Addressing these limitations requires greater attention to device‐level considerations, including optimized, gas‐diffusion electrodes, scalable flow‐cell configurations, and long‐term stability testing under realistic NO_x_ conditions. Advanced in situ and operando characterization techniques will be essential for elucidating the active sites dynamics and catalyst evolution during continuous operation. Overall, Cu‐MXene catalysts nevertheless offer a versatile and promising platform for electrochemical NO_x_ upcycling, bridging pollution control and green NH_3_ production. Continued advances in interface engineering, mechanistic understanding, and system integration are poised to transition this approach beyond the laboratory. These developments will enable high‐efficiency, selective, and scalable NH_3_ synthesis contributing to sustainable, decentralized nitrogen management.

### 6.1 Future Research

Future research should move beyond general catalyst screening and focus on the rational design of well‐defined Cu‐MXene interfaces. Particular attention should be given to controlling Cu dispersion, oxidation state, coordination environment, and metal‐support interaction, as these factors directly influence NOx adsorption, intermediate stabilization, NH_3_ selectivity, and hydrogen evolution suppression. Defect engineering, heteroatom doping, and tailored MXene surface terminations should be systematically investigated to identify which structural features promote the desired NOx‐to‐NH_3_ pathway under realistic operating conditions.

A second priority is to establish clear structure–activity relationships using standardized performance evaluation. Future studies should report NH_3_ yield rate, Faradaic efficiency, partial current density, energy efficiency, product selectivity, and long‐term stability under comparable conditions. Particular care is needed to avoid false‐positive NH_3_ detection, especially in low‐concentration systems, by using rigorous control experiments, isotope‐labeling where applicable, and validated analytical protocols. This will allow meaningful comparison between Cu‐MXene catalysts and other emerging eNOxRR systems.

From a device perspective, future work should shift from conventional H‐type cells toward practical reactor configurations, including gas‐diffusion electrodes, membrane‐electrode assemblies, and flow‐cell systems. These platforms are essential for improving NOx mass transport, increasing current density, and enabling continuous NH_3_ production. Catalyst layers should also be optimized for electrode thickness, binder content, hydrophobicity, gas accessibility, and electrolyte management to prevent flooding, salt accumulation, and catalyst degradation during prolonged operation.

Long‐term durability remains a major challenge. Future studies should evaluate Cu leaching, MXene oxidation, surface reconstruction, loss of conductivity, and changes in active‐site structure during extended operation. In situ and operando techniques, including Raman, FTIR, XAS, EPR, and online product analysis, should be combined with post‐reaction microscopy and spectroscopy to track catalyst evolution and identify the true active phase under reaction conditions.

Finally, techno‐economic and system‐level analysis should become part of future catalyst development. The practical value of Cu‐MXene‐based eNOxRR will depend not only on high activity, but also on catalyst cost, MXene scalability, feedstock availability, NOx concentration, separation of NH_3_ from electrolyte, energy consumption, and integration with renewable electricity. Coupling NOx‐containing waste streams from industrial emissions or wastewater treatment with decentralized electrochemical NH_3_ production could provide a realistic pathway toward pollution control and low‐carbon nitrogen management. Therefore, future progress will require coordinated advances in catalyst design, reactor engineering, mechanistic validation, and process integration.

## Author Contributions

**Gechuanqi Pan**: writing – original draft, methodology, resources, conceptualization. **Asif Mahmood**: writing – review and editing, conceptualization, supervision, validation, project administration. **Hafiz Muhammad Adeel Sharif**: methodology, writing – original draft, writing – review and editing, data curation. **Changping Li**: conceptualization, funding acquisition, writing – review and editing, project administration. **Yuwei Wang**: writing – original draft, data curation, methodology. **Yasir Abbas**: writing – review and editing, validation, supervision.

## Conflicts of Interest

The authors declare no conflicts of interest.

## Data Availability

The data that support the findings of this study are available from the corresponding author upon reasonable request.

## References

[1] S. Li , Y. Zhou , X. Fu , et al., “Long‐term Continuous Ammonia Electrosynthesis,” Nature 629 (2024): 92–97. 38503346 10.1038/s41586-024-07276-5

[2] K. Zhang , A. Cao , L. H. Wandall , et al., “Spin‐mediated Promotion of Co Catalysts for Ammonia Synthesis,” Science 383 (2024): 1357–1363. 38513006 10.1126/science.adn0558

[3] Q. Cheng , A. Muhammad , O. Kaario , Z. Ahmad , and L. Martti , “Ammonia as a Sustainable Fuel: Review and Novel Strategies,” Renewable and Sustainable Energy Reviews 207 (2025): 114995.

[4] S. L. Foster , S. I. P. Bakovic , R. D. Duda , et al., “Catalysts for Nitrogen Reduction to Ammonia,” Nature Catalysis 1 (2018): 490–500.

[5] J. Long , S. Chen , Y. Zhang , et al., “Direct Electrochemical Ammonia Synthesis from Nitric Oxide,” Angewandte Chemie International Edition 59 (2020): 9711–9718. 32189423 10.1002/anie.202002337

[6] G.‐F. Chen , Y. Yuan , H. Jiang , et al., “Electrochemical Reduction of Nitrate to Ammonia via Direct Eight‐electron Transfer Using a Copper–molecular Solid Catalyst,” Nature Energy 5 (2020): 605–613.

[7] L. Wu , J. Feng , L. Zhang , et al., “Boosting Electrocatalytic Nitrate‐to‐Ammonia via Tuning of N‐Intermediate Adsorption on a Zn−Cu Catalyst,” Angewandte Chemie International Edition 62 (2023): 202307952. 10.1002/anie.202307952 37665252

[8] Y. Wang , A. Xu , Z. Wang , et al., “Enhanced Nitrate‐to‐Ammonia Activity on Copper–Nickel Alloys via Tuning of Intermediate Adsorption,” Journal of the American Chemical Society 142 (2020): 5702–5708. 32118414 10.1021/jacs.9b13347

[9] F. Tavares , F. F. Camilo , M. Zbair , L. Limousy , and J. Brendle , “Silver‐Modified Biochar: Investigating NO_2_ Adsorption and Reduction Efficiency at Different Temperatures,” Catalysts 15 (2025): 392.

[10] Y. Wang , W. Zhou , R. Jia , Y. Yu , and B. Zhang , “Unveiling the Activity Origin of a Copper‐Based Electrocatalyst for Selective Nitrate Reduction to Ammonia,” Angewandte Chemie International Edition 59 (2020): 5350–5354. 31965695 10.1002/anie.201915992

[11] Z. Rahman , J. Zhang , L. Zhang , et al., “A Kinetic Evaluation and Optimization Study on NOx Reduction by Reburning under Pressurized Oxy‐combustion,” Journal of Environmental Management 290 (2021): 112690. 33901829 10.1016/j.jenvman.2021.112690

[12] K. Li , S. Z. Andersen , M. J. Statt , et al., “Enhancement of Lithium‐mediated Ammonia Synthesis by Addition of Oxygen,” Science 374 (2021): 1593–1597. 34941415 10.1126/science.abl4300

[13] J. Ding , W. Li , H. Zhang , et al., “A Cascade Jet Plasma Oxidation─Electroreduction System Using Pd‐Ni Dual‐Site Catalyst for Sustainable Ammonia Production from Air,” Advanced Functional Materials 34 (2024): 2410768.

[14] S. Z. Andersen , V. Čolić , S. Yang , et al., “A Rigorous Electrochemical Ammonia Synthesis Protocol with Quantitative Isotope Measurements,” Nature 570 (2019): 504–508. 31117118 10.1038/s41586-019-1260-x

[15] B. Gurkan , X. Su , A. Klemm , et al., “Perspective and Challenges in Electrochemical Approaches for Reactive CO_2_ Separations,” iScience 24 (2021): 103422. 34877489 10.1016/j.isci.2021.103422 PMC8633013

[16] H. R. Inta , D. Dhanabal , S. S. Markandaraj , and S. Shanmugam , “Recent Advances in Electrocatalytic NO_X_ Reduction into Ammonia,” EES Catalysis 1 (2023): 645–664.

[17] J. Theerthagiri , K. Karuppasamy , A. H. Mahadi , et al., “Electrochemical Reduction of Gaseous Nitric Oxide into Ammonia: a Review,” Environmental Chemistry Letters 22 (2024): 189–208.

[18] J. Zhang , J. Liu , Q. Peng , X. Wang , and Y. Li , “Nearly Monodisperse Cu_2_O and CuO Nanospheres: Preparation and Applications for Sensitive Gas Sensors,” Chemistry of Materials 18 (2006): 867–871.

[19] Y. Wang , S. Rahimnejad , W.‐J. Sun , et al., “Bimetallic Cu‐Fe Catalysts on MXene for Synergistically Electrocatalytic Conversion of Nitrate to Ammonia,” Journal of Colloid and Interface Science 648 (2023): 595–603. 37315481 10.1016/j.jcis.2023.06.008

[20] Y. Cheng , X. Li , P. Shen , Y. Guo , and K. Chu , “MXene Quantum Dots/Copper Nanocomposites for Synergistically Enhanced N_2_ Electroreduction,” Energy & Environmental Materials 6 (2023): 12268.

[21] Y. Wang , W. Zhang , W. Wen , et al., “Atomically Dispersed Unsaturated Cu‐N_3_ Sites on High‐Curvature Hierarchically Porous Carbon Nanotube for Synergetic Enhanced Nitrate Electroreduction to Ammonia,” Advanced Functional Materials 33 (2023): 2302651.

[22] K. Chen , J. Xiang , Y. Guo , X. Liu , X. Li , and K. Chu , “Pd 1 Cu Single‐Atom Alloys for High‐Current‐Density and Durable NO‐to‐NH_3_ Electroreduction,” Nano Letters 24 (2024): 541–548. 38185876 10.1021/acs.nanolett.3c02259

[23] H. M. A. Sharif , J. Huang , A. Mahmood , et al., “Optimized Electron Transfer in Ru/Cu@MXene Electrocatalysts for Ultra‐selective and High‐yield Electrochemical NH_3_ Synthesis,” Nano Materials Science (2025), 10.1016/j.nanoms.2025.10.014.

[24] L. Li , S. Gu , S. Guo , et al., “MXenes as Multifunctional Catalysts in Fenton‐like Reactions for Water Purification: Mechanisms, Applications, and Perspectives,” Journal of Environmental Management 399 (2026): 128532. 41548549 10.1016/j.jenvman.2025.128532

[25] Z. Bo , M. Cao , Y. Wang , J. Yan , K. Cen , and X. Tu , “Unlocking the Potential of Cu/Ti_3_C_2_Tx MXene Catalyst in Plasma Catalytic CO_2_ Hydrogenation,” Journal of the Energy Institute 115 (2024): 101648.

[26] J. Wang , T. Feng , J. Chen , J.‐H. He , and X. Fang , “Flexible 2D cu Metal: Organic Framework@ MXene Film Electrode with Excellent Durability for Highly Selective Electrocatalytic NH_3_ Synthesis,” Research 2022 (2022): 9837012. 35707045 10.34133/2022/9837012 PMC9175116

[27] R. Nittoor‐Veedu , B. Kalleshappa , and M. Pumera , “Copper‐Infused MXene from MAX Phase for Enhanced Electrochemical Ammonia Production,” ACS Nano 19 (2025): 39645–39653. 41223408 10.1021/acsnano.5c09659 PMC12659423

[28] H. M. Adeel Sharif , M. Rashad , I. Hussain , A. Abbas , O. F. Aldosari , and C. Li , “Green Energy Harvesting from CO_2_ and NOx by MXene Materials: Detailed Historical and Future Prospective,” Applied Catalysis B: Environment and Energy 344 (2024): 123585.

[29] W. Yang , H. Liu , X. Chang , et al., “Electrosynthesis of NH_3_ from NO with Ampere‐level Current Density in a Pressurized Electrolyzer,” Nature Communications 16 (2025): 1257. 10.1038/s41467-025-56548-9 PMC11787339 39893185

[30] Y.‐Z. Yu , Y. Cheng , S. Cheng , and Z.‐Y. Wu , “Advanced Ruthenium‐Based Electrocatalysts for NO_X_ Reduction to Ammonia,” Advanced Materials 37 (2025): 2412363. 10.1002/adma.202412363 39676485

[31] F. Zhao , G. Li , Q. Hua , et al., “A Two‐dimensional MXene‐supported CuRu Catalyst for Efficient Electrochemical Nitrate Reduction to Ammonia,” Catalysis Science & Technology 13 (2023): 5543–5548.

[32] H. Shen , C. Choi , J. Masa , et al., “Electrochemical Ammonia Synthesis: Mechanistic Understanding and Catalyst Design,” Chemistry 7 (2021): 1708–1754.

[33] A. R. Singh , B. A. Rohr , J. A. Schwalbe , et al., “Electrochemical Ammonia Synthesis—The Selectivity Challenge,” ACS Catalysis 7 (2017): 706–709.

[34] J. Linnemann , K. Kanokkanchana , and K. Tschulik , “Design Strategies for Electrocatalysts from an Electrochemist's Perspective,” ACS Catalysis 11 (2021): 5318–5346.

[35] C. Bai , S. Fan , X. Li , et al., “Hollow Cu_2_O@CoMn_2_O_4_ Nanoreactors for Electrochemical NO Reduction to NH_3_: Elucidating the Void‐Confinement Effects on Intermediates,” Advanced Functional Materials 32 (2022): 2205569.

[36] J. Shi , C. Wang , R. Yang , et al., “Promoting Nitric Oxide Electroreduction to Ammonia over Electron‐rich Cu Modulated by Ru Doping,” Science China Chemistry 64 (2021): 1493–1497.

[37] J.‐J. Zhang , Y.‐Y. Lou , Z. Wu , X. J. Huang , and S.‐G. Sun , “Spatially Separated Cu/Ru on Ordered Mesoporous Carbon for Superior Ammonia Electrosynthesis from Nitrate over a Wide Potential Window,” Journal of the American Chemical Society 146 (2024): 24966–24977. 39197103 10.1021/jacs.4c06657

[38] X. Yang , R. Wang , S. Wang , et al., “Sequential Active‐site Switches in Integrated Cu/Fe‐TiO_2_ for Efficient Electroreduction from Nitrate into Ammonia,” Applied Catalysis B: Environmental 325 (2023): 122360.

[39] S. Ingavale , P. Marbaniang , M. Palabathuni , and N. Mishra , “In Situ Growth of Copper Oxide on MXene by Combustion Method for Electrochemical Ammonia Production from Nitrate,” Nanoscale Advances 6 (2024): 481–488. 38235088 10.1039/d3na00609c PMC10791130

[40] H. M. A. Sharif , H. M. F. Khan , S. Ullah , et al., “Progress in Electrocatalytic Nitrate Reduction for Green Energy: Catalyst Engineering, Mechanisms, and Techno‐economic Feasibility,” Journal of Energy Chemistry 95 (2024): 380–406.

[41] H. Niu , Z. Zhang , X. Wang , X. Wan , C. Shao , and Y. Guo , “Theoretical Insights into the Mechanism of Selective Nitrate‐to‐Ammonia Electroreduction on Single‐Atom Catalysts,” Advanced Functional Materials 31 (2021): 2008533.

[42] J. Shao , H. Jing , P. Wei , et al., “Electrochemical Synthesis of Ammonia from Nitric Oxide Using a Copper–tin Alloy Catalyst,” Nature Energy 8 (2023): 1273–1283.

[43] B. H. Ko , B. Hasa , H. Shin , Y. Zhao , and F. Jiao , “Electrochemical Reduction of Gaseous Nitrogen Oxides on Transition Metals at Ambient Conditions,” Journal of the American Chemical Society 144 (2022): 1258–1266. 35014265 10.1021/jacs.1c10535

[44] S. Cheon , W. J. Kim , D. Y. Kim , Y. Kwon , and J.‐I. Han , “Electro‐synthesis of Ammonia from Dilute Nitric Oxide on a Gas Diffusion Electrode,” ACS Energy Letters 7 (2022): 958–965.

[45] A. Hermawan , V. N. Alviani , and Z. W. Seh , “Fundamentals, Rational Catalyst Design, and Remaining Challenges in Electrochemical NOx Reduction Reaction,” iScience 26 (2023): 107410. 37593457 10.1016/j.isci.2023.107410 PMC10428125

[46] J. Zhou , Y. Zhu , K. Wen , et al., “Efficient and Selective Electrochemical Nitrate Reduction to N_2_ Using a Flow‐Through Zero‐Gap Electrochemical Reactor with a Reconstructed Cu(OH)_2_ Cathode: Insights into the Importance of Inter‐Electrode Distance,” Environmental Science & Technology 58 (2024): 4824–4836. 38408018 10.1021/acs.est.3c10936

[47] J. Sun , Y. Lv , H. Chang , H. Ou , Y. Li , and G. Yang , “Modeling of Three‐dimensional Flow Cell Reactor with Serpentine Channel for Nitrate Electroreduction to Ammonia,” Chemical Engineering Science 315 (2025): 121845.

[48] Y. Lv , J. Chen , P. Bai , et al., “Mass Transfer and Electrochemical Behavior of Nitrate Reduction to Ammonia in Electrocatalytic Flow Cell Reactor,” AIChE Journal 70 (2024): 18262.

[49] M. Riyaz and A. Bagger , “NO_X_ Reduction Mechanism: Thermal vs Electrochemical Step,” Electrochimica Acta 513 (2025): 145429.

[50] L. Zhang , L.‐X. Ding , G.‐F. Chen , X. Yang , and H. Wang , “Ammonia Synthesis under Ambient Conditions: Selective Electroreduction of Dinitrogen to Ammonia on Black Phosphorus Nanosheets,” Angewandte Chemie International Edition 58 (2019): 2612–2616. 30560583 10.1002/anie.201813174

[51] A. Liu , X. Liang , Q. Yang , et al., “Electrocatalytic Synthesis of Ammonia Using a 2D Ti_3_C_2_ MXene Loaded with Copper Nanoparticles,” ChemPlusChem 86 (2021): 166–170. 33215874 10.1002/cplu.202000702

[52] S. Ali , S. Ali , A. Ismail , M. Zahid , F. Raziq , and L. Qiao , “Cu‐based Metal Oxide Catalysts for NH_3_‐SCR of NO: from Fundamentals to Mechanistic Insights,” Coordination Chemistry Reviews 536 (2025): 216676.

[53] J. Deng , S. Wang , T. Lan , S. Guo , K. Zhang , and D. Zhang , “Precise Regeneration of NOx Reduction Catalysts Poisoned by Metal Ions via Sabatier Principle of Antidote‐active Center Interaction,” Journal of Cleaner Production 417 (2023): 137967.

[54] Y. Shen , W. Dong , L. Zhang , et al., “Revealing the Mechanism of K‐enhanced Cu‐SSZ‐13 Catalysts against Hydrothermal Aging and P‐poisoning for NOx Reduction by NH3‐SCR,” Separation and Purification Technology 330 (2024): 125248.

[55] J. Huang , H. M. A. Sharif , A. Mahmood , and C. Li , “Unveiling the Heterojunction of Fe_3_O_4_@MXene Nanocatalyst for Ultrafast Degradation of Antibiotics via Non‐radical Pathway,” Chemical Engineering Journal 500 (2024): 156941.

[56] Y. Wang , J. Li , and Z. Liu , “Selective Catalytic Reduction of NOx by NH_3_ over Cu‐AEI Zeolite Catalyst: Current Status and Future Perspectives,” Applied Catalysis B: Environmental 343 (2024): 123479.

[57] N. Zhu , W. Shan , Y. Shan , et al., “Effects of Alkali and Alkaline Earth Metals on Cu‐SSZ‐39 Catalyst for the Selective Catalytic Reduction of NO with NH_3_ ,” Chemical Engineering Journal 388 (2020): 124250.

[58] H. Xue , X. Guo , S. Wang , C. Sun , J. Yu , and D. Mao , “Poisoning Effect of CaO on Cu/ZSM‐5 for the Selective Catalytic Reduction of NO with NH_3_ ,” Catalysis Communications 112 (2018): 53–57.

[59] K. Chen , G. Zhang , X. Li , X. Zhao , and K. Chu , “Electrochemical NO Reduction to NH_3_ on Cu Single Atom Catalyst,” Nano Research 16 (2023): 5857–5863.

[60] S. Wee , X. Lian , E. Vorobyeva , et al., “Tuning MXene Properties through Cu Intercalation: Coupled Guest/Host Redox and Pseudocapacitance,” ACS Nano 18 (2024): 10124–10132. 38511608 10.1021/acsnano.3c12989 PMC11008361

[61] N. Dutta , D. Bagchi , G. Chawla , and S. C. Peter , “A Guideline to Determine Faradaic Efficiency in Electrochemical CO_2_ Reduction,” ACS Energy Letters 9 (2024): 323–328.

[62] C. Wei , R. R. Rao , J. Peng , et al., “Recommended Practices and Benchmark Activity for Hydrogen and Oxygen Electrocatalysis in Water Splitting and Fuel Cells,” Advanced Materials 31 (2019): 1806296. 10.1002/adma.201806296 30656754

[63] Z. Zhou , Z. Pei , L. Wei , S. Zhao , X. Jian , and Y. Chen , “Electrocatalytic Hydrogen Evolution under Neutral pH Conditions: Current Understandings, Recent Advances, and Future Prospects,” Energy & Environmental Science 13 (2020): 3185–3206.

[64] G. Qing , R. Ghazfar , S. T. Jackowski , et al., “Recent Advances and Challenges of Electrocatalytic N_2_ Reduction to Ammonia,” Chemical Reviews 120 (2020): 5437–5516. 32459470 10.1021/acs.chemrev.9b00659

[65] D. R. MacFarlane , P. V. Cherepanov , J. Choi , et al., “A Roadmap to the Ammonia Economy,” Joule 4 (2020): 1186–1205.

[66] S. Wang , J. Xia , X. Yang , et al., “Two‐dimensional Materials for NOx Reduction to Ammonia: from Electrocatalyst to System,” Coordination Chemistry Reviews 535 (2025): 216610.

[67] Z. Cui , C. Li , W. Peng , and J. Liu , “Emerging MXene‐based Electrocatalysts for Efficient Nitrate Reduction to Ammonia: Recent Advance, Challenges, and Prospects,” Energy Materials 4 (2024): 400057.

[68] Y. Tan , Y. Zhao , X. Chen , et al., “Cooperative Cu with Defective MXene for Enhanced Nitrate Electroreduction to Ammonia,” EcoEnergy 2 (2024): 258–267.

[69] Y. Bai , C. Liu , T. Chen , et al., “MXene‐Copper/Cobalt Hybrids via Lewis Acidic Molten Salts Etching for High Performance Symmetric Supercapacitors,” Angewandte Chemie International Edition 60 (2021): 25318–25322. 34585486 10.1002/anie.202112381

[70] L. Liu , Y. Zhao , X. Lin , C. Ren , Y. Gao , and W. Zhu , “Ammonia Synthesis via Nitric Oxide Electrochemical Reduction on OH‐MXenes: a Universal Descriptor,” The Journal of Physical Chemistry C 129 (2025): 4067–4076.

[71] Y. Hua and L. Zhang , “Single‐atomic Cu Sites for High‐efficiency Electrochemical Ammonia Synthesis from Nitrate,” Molecular Catalysis 567 (2024): 114433.

[72] L. Shan , Y. Ma , S. Xu , et al., “Efficient Electrochemical Reduction of Nitrate to Ammonia over Metal‐organic Framework Single‐atom Catalysts,” Communications Materials 5 (2024): 104.

[73] L. Kong , M. Wang , and C.‐M. L. Wu , “From Single Atom to Low‐Nuclearity Cluster Immobilized Ti_3_C_2_T_X_ MXene for Highly Efficient NO Electroreduction to NH_3_ ,” ACS Materials Letters 6 (2024): 1711–1721.

[74] X. Chen , Y. Cheng , B. Zhang , J. Zhou , and S. He , “Gradient‐concentration RuCo Electrocatalyst for Efficient and Stable Electroreduction of Nitrate into Ammonia,” Nature Communications 15 (2024): 6278. 10.1038/s41467-024-50670-w PMC11272931 39054325

[75] S. Zhang , J. Wu , M. Zheng , et al., “Fe/Cu Diatomic Catalysts for Electrochemical Nitrate Reduction to Ammonia,” Nature Communications 14 (2023): 3634. 10.1038/s41467-023-39366-9 PMC10279643 37337012

[76] W. Luo , J. Liu , Y. Hu , and Q. Yan , “Single and Dual‐atom Catalysts towards Electrosynthesis of Ammonia and Urea: a Review,” Nanoscale 16 (2024): 20463–20483. 39435616 10.1039/d4nr02387k

[77] B. Li , Y. Zhu , and W. Guo , “Recent Advances of Metal Oxide Catalysts for Electrochemical NH_3_ Production from Nitrogen‐containing Sources,” Inorganic Chemistry Frontiers 10 (2023): 5812–5838.

[78] T. Jin , J. Wang , Y. Gong , et al., “Mechanochemical‐tuning Size Dependence of Iridium Single Atom and Nanocluster toward Highly Selective Ammonium Production,” Chem Catalysis 3 (2023): 100477.

[79] X. Zhan , C. Si , J. Zhou , and Z. Sun , “MXene and MXene‐based Composites: Synthesis, Properties and Environment‐related Applications,” Nanoscale Horizons 5 (2020): 235–258.

[80] Y. Xiong , Y. Wang , J. Zhou , F. Liu , F. Hao , and Z. Fan , “Electrochemical Nitrate Reduction: Ammonia Synthesis and the Beyond,” Advanced Materials 36 (2024): 2304021. 10.1002/adma.202304021 37294062

[81] J. Zhang , H. Tan , G. Zhang , and G. Li , “NOx Removal Capacity and Compressive Strength of Photocatalytic Cementitious Materials with Various Color Properties,” Journal of Environmental Management 356 (2024): 120605. 38498962 10.1016/j.jenvman.2024.120605

[82] K. S. Aswathi , K. Unni , S. Mukhopadhyay , et al., “Ambient Microdroplet Synthesis of Pt and Pt–Cu Nanorods from Homogeneous Solutions for Electrocatalytic Nitrate Reduction,” Nanoscale Horizons 11 (2026): 1053–1062. 41574540 10.1039/d5nh00572h

[83] H. Wan , A. Bagger , and J. Rossmeisl , “Electrochemical Nitric Oxide Reduction on Metal Surfaces,” Angewandte Chemie International Edition 60 (2021): 21966–21972. 34350689 10.1002/anie.202108575

[84] J. Feng , H. Tang , H. Guo , and A. Ding , “Visible‐light‐induced Dehydrogenation of Dihydroquinolinones via the Combination of Energy Transfer and Hydrogen Atom Transfers,” Organic Chemistry Frontiers 12 (2025): 1183–1188.

[85] Y. Liu , Y. Luo , Q. Li , J. Wang , and K. Chu , “Bimetallic MnMoO_4_ with Dual‐active‐centers for Highly Efficient Electrochemical N_2_ Fixation,” Chemical Communications 56 (2020): 10227–10230. 32749406 10.1039/d0cc04340k

[86] Y. Cheng , H. Nan , Q. Li , Y. Luo , and K. Chu , “A Rare‐Earth Samarium Oxide Catalyst for Electrocatalytic Nitrogen Reduction to Ammonia,” ACS Sustainable Chemistry & Engineering 8 (2020): 13908–13914.

[87] Y. Lee , S. J. Kim , Y.‐J. Kim , et al., “Oxidation‐resistant Titanium Carbide MXene Films,” Journal of Materials Chemistry A 8 (2020): 573–581.

[88] H. Xu , J. Wu , W. Luo , Q. Li , W. Zhang , and J. Yang , “Dendritic Cell‐Inspired Designed Architectures toward Highly Efficient Electrocatalysts for Nitrate Reduction Reaction,” Small 16 (2020): 2001775. 10.1002/smll.202001775 32583581

[89] R. Zhang , S. Zhang , H. Cui , Y. Guo , N. Li , and C. Zhi , “Electrochemical Nitrate Reduction to Ammonia Using Copper‐based Electrocatalysts,” Next Energy 4 (2024): 100125.

[90] L.‐X. Li , W.‐J. Sun , H.‐Y. Zhang , et al., “Highly Efficient and Selective Nitrate Electroreduction to Ammonia Catalyzed by Molecular Copper Catalyst@Ti_3_C_2_T_X_ MXene,” Journal of Materials Chemistry A 9 (2021): 21771–21778.

[91] X. Yang , G. Wei , J. Cao , et al., “Mxene‐Cu Electrified Membranes with Confined Lamellar Channels for the Flow‐through Electrochemical Reduction of Nitrate to Ammonia,” ACS Sustainable Chemistry & Engineering 12 (2024): 3378–3389.

[92] S. Ajmal , A. Kumar , M. Selvaraj , et al., “MXenes and Their Interfaces for the Taming of Carbon Dioxide & Nitrate: a Critical Review,” Coordination Chemistry Reviews 483 (2023): 215094.

[93] J. Jia , Z. Hou , N. He , L. Cai , and J. Hui , “Fabrication, Microstructure and Properties of Ti_3_C_2_Tx MXene Nanosheets Reinforced Cu Composites,” Journal of Materials Research and Technology 23 (2023): 503–514.

[94] Y. Yang , Y. Xu , Q. Li , Y. Zhang , and H. Zhou , “Two‐dimensional Carbide/Nitride (MXene) Materials in Thermal Catalysis,” Journal of Materials Chemistry A 10 (2022): 19444–19465.

[95] Y. Wang , C. Wang , M. Li , Y. Yu , and B. Zhang , “Nitrate Electroreduction: Mechanism Insight, in Situ Characterization, Performance Evaluation, and Challenges,” Chemical Society Reviews 50 (2021): 6720–6733. 33969861 10.1039/d1cs00116g

[96] H. Liu , X. Lang , C. Zhu , et al., “Efficient Electrochemical Nitrate Reduction to Ammonia with Copper‐Supported Rhodium Cluster and Single‐Atom Catalysts,” Angewandte Chemie International Edition 61 (2022): 202202556. 10.1002/anie.202202556 35297151

[97] G. Mestl , “In Situ Raman Spectroscopy — A Valuable Tool to Understand Operating Catalysts,” Journal of Molecular Catalysis A: Chemical 158 (2000): 45–65.

[98] M. Delhaye and P. Dhamelincourt , “Raman Microprobe and Microscope with Laser Excitation,” Journal of Raman Spectroscopy 3 (1975): 33–43.

[99] P. M. Radjenovic , R.‐Y. Zhou , J.‐C. Dong , and J.‐F. Li , “Watching Reactions at Solid–Liquid Interfaces with in Situ Raman Spectroscopy,” The Journal of Physical Chemistry C 125 (2021): 26285–26295.

[100] G. Demirel , H. Usta , M. Yilmaz , M. Celik , H. A. Alidagi , and F. Buyukserin , “Surface‐enhanced Raman Spectroscopy (SERS): an Adventure from Plasmonic Metals to Organic Semiconductors as SERS Platforms,” Journal of Materials Chemistry C 6 (2018): 5314–5335.

[101] W. He , J. Zhang , S. Dieckhöfer , et al., “Splicing the Active Phases of Copper/Cobalt‐based Catalysts Achieves High‐rate Tandem Electroreduction of Nitrate to Ammonia,” Nature Communications 13 (2022): 1129. 10.1038/s41467-022-28728-4 PMC8891333 35236840

[102] Y. Zhao , X. Chang , A. S. Malkani , et al., “Speciation of Cu Surfaces during the Electrochemical CO Reduction Reaction,” Journal of the American Chemical Society 142 (2020): 9735–9743. 32338904 10.1021/jacs.0c02354

[103] Y. Liu , B. Deng , K. Li , H. Wang , Y. Sun , and F. Dong , “Metal‐organic Framework Derived Carbon‐supported Bimetallic Copper‐nickel Alloy Electrocatalysts for Highly Selective Nitrate Reduction to Ammonia,” Journal of Colloid and Interface Science 614 (2022): 405–414. 35108632 10.1016/j.jcis.2022.01.127

[104] H. Chen , H. Li , Y. Zhao , et al., “Enhancing NO3− Reduction on BiFeO_3_/Cu Cathode via Ferroelectric Polarization: a Novel Strategy for Surface Electronic Structure Regulation,” Journal of Environmental Management 394 (2025): 127380. 40986954 10.1016/j.jenvman.2025.127380

[105] T. Zhang , J. Lv , R. Yang , et al., “Boosting Electrochemical Ammonia Synthesis via NO_X_ Reduction over Sulfur‐Doped Copper Oxide Nanoneedle Arrays,” Advanced Energy Materials 14 (2024): 2400790.

[106] Y. Wang , D. Chao , Z. Wang , J. Ni , and L. Li , “An Energetic CuS–Cu Battery System Based on CuS Nanosheet Arrays,” ACS Nano 15 (2021): 5420–5427. 33709698 10.1021/acsnano.1c00075

[107] R. Daiyan , T. Tran‐Phu , P. Kumar , et al., “Nitrate Reduction to Ammonium: from CuO Defect Engineering to Waste NO_X_‐to‐NH_3_ Economic Feasibility,” Energy & Environmental Science 14 (2021): 3588–3598.

[108] L. Magagnin , R. Maboudian , and C. Carraro , “Gold Deposition by Galvanic Displacement on Semiconductor Surfaces: Effect of Substrate on Adhesion,” The Journal of Physical Chemistry B 106 (2002): 401–407.

[109] G. Ramer and B. Lendl , “Attenuated Total Reflection Fourier Transform Infrared Spectroscopy,” Encyclopedia of Analytical Chemistry 15 (2013): 1–27.

[110] S. G. Kazarian and K. L. A. Chan , “ATR‐FTIR Spectroscopic Imaging: Recent Advances and Applications to Biological Systems,” The Analyst 138 (2013): 1940–1951. 23400222 10.1039/c3an36865c

[111] J.‐Y. Ye , Y.‐X. Jiang , T. Sheng , and S.‐G. Sun , “In‐situ FTIR Spectroscopic Studies of Electrocatalytic Reactions and Processes,” Nano Energy 29 (2016): 414–427.

[112] Y. Deng , S. Dong , Z. Li , H. Jiang , X. Zhang , and X. Ji , “Applications of Conventional Vibrational Spectroscopic Methods for Batteries beyond Li‐Ion,” Small Methods 2 (2018): 1700332.

[113] S. Han , H. Li , T. Li , et al., “Ultralow Overpotential Nitrate Reduction to Ammonia via a Three‐step Relay Mechanism,” Nature Catalysis 6 (2023): 402–414.

[114] A. D. Handoko , S. N. Steinmann , and Z. W. Seh , “Theory‐guided Materials Design: Two‐dimensional MXenes in Electro‐ and Photocatalysis,” Nanoscale Horizons 4 (2019): 809–827.

[115] X. Lei , Q. Tang , Y. Zheng , et al., “High‐entropy Single‐atom Activated Carbon Catalysts for Sustainable Oxygen Electrocatalysis,” Nature Sustainability 6 (2023): 816–826.

[116] R. Zhang , Y. Zhang , B. Xiao , et al., “Phase Engineering of High‐Entropy Alloy for Enhanced Electrocatalytic Nitrate Reduction to Ammonia,” Angewandte Chemie International Edition 63 (2024): 202407589. 10.1002/anie.202407589 38703065

[117] Q. Hu , K. Yang , O. Peng , et al., “Ammonia Electrosynthesis from Nitrate Using a Ruthenium–Copper Cocatalyst System: a Full Concentration Range Study,” Journal of the American Chemical Society 146 (2024): 668–676. 38154089 10.1021/jacs.3c10516

[118] E. Groppo , S. Rojas‐Buzo , and S. Bordiga , “The Role of in Situ / Operando IR Spectroscopy in Unraveling Adsorbate‐Induced Structural Changes in Heterogeneous Catalysis,” Chemical Reviews 123 (2023): 12135–12169. 37882638 10.1021/acs.chemrev.3c00372 PMC10636737

[119] S. A. Bonke , T. Risse , A. Schnegg , and A. Brückner , “In Situ Electron Paramagnetic Resonance Spectroscopy for Catalysis,” Nature Reviews Methods Primers 1 (2021): 33.

[120] R. Escudero , J. Segura , R. Velasco , et al., “Electron Spin Resonance (ESR) Spectroscopy Study of Cheese Treated with Accelerated Electrons,” Food Chemistry 276 (2019): 315–321. 30409600 10.1016/j.foodchem.2018.09.101

[121] K. Wang , R. Mao , R. Liu , et al., “Intentional Corrosion‐induced Reconstruction of Defective NiFe Layered Double Hydroxide Boosts Electrocatalytic Nitrate Reduction to Ammonia,” Nature Water 1 (2023): 1068–1078.

[122] S. V. Yurtaeva , I. F. Gilmutdinov , A. A. Rodionov , et al., “Ferromagnetically Coupled Copper(II) Clusters Incorporated in Functionalized Boltorn H30 Hyperbranched Polymer Architecture: ESR, Magnetic Susceptibility Measurements, and Quantum‐Chemical Calculations,” ACS Omega 4 (2019): 16450–16461. 31616823 10.1021/acsomega.9b02048 PMC6787908

[123] B. Zhou , Q. Liu , L. Shi , and Z. Liu , “Electron Spin Resonance Studies of Coals and Coal Conversion Processes: a Review,” Fuel Processing Technology 188 (2019): 212–227.

[124] X. Li , S. Wang , L. Li , Y. Sun , and Y. Xie , “Progress and Perspective for in Situ Studies of CO_2_ Reduction,” Journal of the American Chemical Society 142 (2020): 9567–9581. 32357008 10.1021/jacs.0c02973

[125] A. Prajapati , C. Hahn , I. M. Weidinger , et al., “Best Practices for in‐situ and Operando Techniques within Electrocatalytic Systems,” Nature Communications 16 (2025): 2593. 10.1038/s41467-025-57563-6 PMC11911412 40091111

[126] J. Lim , D. A. Cullen , E. Stavitski , S. W. Lee , and M. C. Hatzell , “Atomically Ordered PdCu Electrocatalysts for Selective and Stable Electrochemical Nitrate Reduction,” ACS Energy Letters 8 (2023): 4746–4752. 37969250 10.1021/acsenergylett.3c01672 PMC10644382

[127] A. Boubnov , H. W. P. Carvalho , D. E. Doronkin , et al., “Selective Catalytic Reduction of NO over Fe‐ZSM‐5: Mechanistic Insights by Operando HERFD‐XANES and Valence‐to‐Core X‐ray Emission Spectroscopy,” Journal of the American Chemical Society 136 (2014): 13006–13015. 25105343 10.1021/ja5062505

[128] T. Günter , D. E. Doronkin , A. Boubnov , H. W. P. Carvalho , M. Casapu , and J. D. Grunwaldt , “The SCR of NOx with NH_3_ Examined by Novel X‐ray Emission and X‐ray Absorption Methods,” Topics in Catalysis 59 (2016): 866–874.

[129] U. Bergmann and P. Glatzel , “X‐ray Emission Spectroscopy,” Photosynthesis Research 102 (2009): 255–266. 19705296 10.1007/s11120-009-9483-6

[130] J. Timoshenko and B. R. Cuenya , “In Situ/Operando Electrocatalyst Characterization by X‐ray Absorption Spectroscopy,” Chemical reviews 121 (2021): 882–961. 32986414 10.1021/acs.chemrev.0c00396 PMC7844833

[131] T. Chankhanittha , T. Butburee , N. Punklahan , A. Jiratanachotikul , K. Wannakan , and P. Khemthong , “Metallic Foam‐mediated Thermal Exfoliation for Cu Single‐atom Anchoring on G‐C_3_N_4_ via Cu‐N_2_ Coordination toward Efficient CO_2_ Photoreduction,” Journal of Environmental Chemical Engineering 14 (2026): 121610.

[132] Y. Zeng , X. Qi , S. Lu , M. N. Khalil , X. Dong , and H. Wang , “Defect Engineering of Nickel‐Based Compounds for Energy‐Saving H_2_ Production,” Energies 17 (2024): 3801.

[133] B. Dziejarski , J. Serafin , K. Andersson , and R. Krzyżyńska , “CO_2_ Capture Materials: a Review of Current Trends and Future Challenges,” Mater Today Sustain 24 (2023): 100483.

[134] E.‐G. Han , J.‐H. Lee , and M.‐S. Kang , “ZIF‐8‐Embedded Cation‐Exchange Membranes with Improved Monovalent Ion Selectivity for Capacitive Deionization,” Membranes 15 (2025): 19. 39852260 10.3390/membranes15010019 PMC11766747

[135] P. P. Lopes , D. Li , H. Lv , et al., “Eliminating Dissolution of Platinum‐based Electrocatalysts at the Atomic Scale,” Nature Materials 19 (2020): 1207–1214. 32690912 10.1038/s41563-020-0735-3

[136] J. Zhang , Y. Yuan , L. Gao , G. Zeng , M. Li , and H. Huang , “Stabilizing Pt‐Based Electrocatalysts for Oxygen Reduction Reaction: Fundamental Understanding and Design Strategies,” Advanced Materials 33 (2021): 2006494. 10.1002/adma.202006494 33825222

[137] M. G. Daniel , J. Song , S. Ali Safiabadi Tali , X. Dai , and W. Zhou , “Sub‐10 Nm Nanolaminated Al_2_O_3_/HfO_2_ Coatings for Long‐Term Stability of Cu Plasmonic Nanodisks in Physiological Environments,” ACS Applied Materials & Interfaces 12 (2020): 31952–31961. 32544317 10.1021/acsami.0c06941

[138] Y. Ren , C. Yu , X. Tan , H. Huang , Q. Wei , and J. Qiu , “Strategies to Suppress Hydrogen Evolution for Highly Selective Electrocatalytic Nitrogen Reduction: Challenges and Perspectives,” Energy & Environmental Science 14 (2021): 1176–1193.

[139] Y. Wang , H. Yin , F. Dong , et al., “N‐Coordinated Cu–Ni Dual‐Single‐Atom Catalyst for Highly Selective Electrocatalytic Reduction of Nitrate to Ammonia,” Small 19 (2023): 2207695. 10.1002/smll.202207695 36793161

[140] P. O. Å. Persson and J. Rosen , “Current state of the Art on Tailoring the MXene Composition, Structure, and Surface Chemistry,” Current Opinion in Solid State and Materials Science 23 (2019): 100774.

[141] T. Su , X. Ma , J. Tong , H. Ji , Z. Qin , and Z. Wu , “Surface Engineering of MXenes for Energy and Environmental Applications,” Journal of Materials Chemistry A 10 (2022): 10265–10296.

[142] D. Yin , Y. Liu , P. Song , et al., “In Situ Growth of Copper/Reduced Graphene Oxide on Graphite Surfaces for the Electrocatalytic Reduction of Nitrate,” Electrochimica Acta 324 (2019): 134846.

[143] S. Zhang , J. Geng , Z. Zhao , et al., “High‐efficiency Electrosynthesis of Urea over Bacterial Cellulose Regulated Pd–Cu Bimetallic Catalyst,” EES Catalysis 1 (2023): 45–53.

[144] X. Guo , J. Yu , S. Ren , R.‐T. Gao , L. Wu , and L. Wang , “Controlled Defective Engineering on CuIr Catalyst Promotes Nitrate Selective Reduction to Ammonia,” ACS Nano 18 (2024): 24252–24261. 39169609 10.1021/acsnano.4c05772

[145] L. Fang , S. Lu , S. Wang , et al., “Defect Engineering on Electrocatalysts for Sustainable Nitrate Reduction to Ammonia: Fundamentals and Regulations,” Chemistry–A European Journal 30 (2024): 202303249. 10.1002/chem.202303249 37997008

[146] Y. Wang , X. Zhu , Q. An , et al., “Electron Deficiency Is More Important than Conductivity in C−N Coupling for Electrocatalytic Urea Synthesis,” Angewandte Chemie International Edition 63 (2024): 202410938. 10.1002/anie.202410938 39092496

[147] A. Paliwal , C. D. Bandas , E. S. Thornburg , R. T. Haasch , and A. A. Gewirth , “Enhanced Nitrate Reduction Activity from Cu‐Alloy Electrodes in an Alkaline Electrolyte,” ACS Catalysis 13 (2023): 6754–6762.

[148] H. Huang , Y. Zhang , W. Chen , et al., “Dynamic Stabilization of Cu^δ+^ in Heterostructured Ag^0^‐CuAgO X for High‐Performing Nitrate Electroreduction,” Advanced Energy Materials 15 (2025): 2405534.

[149] L. Jiang , X. Bai , X. Zhi , and Y. Jiao , “New Mechanistic Insights into Electrokinetic Competition between Nitrogen Reduction and Hydrogen Evolution Reactions,” Advanced Energy Materials 14 (2024): 2303809.

[150] R. Thakur , A. VahidMohammadi , J. Moncada , et al., “Insights into the Thermal and Chemical Stability of Multilayered V_2_CT_X_ MXene,” Nanoscale 11 (2019): 10716–10726. 31120085 10.1039/c9nr03020d

[151] M. J. B. Kabeyi and O. A. Olanrewaju , “Sustainable Energy Transition for Renewable and Low Carbon Grid Electricity Generation and Supply,” Frontiers in Energy Research 9 (2022): 743114.

